# Recent Progress in Metal-Organic Framework Based Fluorescent Sensors for Hazardous Materials Detection

**DOI:** 10.3390/molecules27072226

**Published:** 2022-03-29

**Authors:** Dan Zhao, Shuang Yu, Wen-Jie Jiang, Zhi-Hao Cai, Dan-Li Li, Ya-Lan Liu, Zhi-Zhou Chen

**Affiliations:** 1 School of Marine Science, Ningbo University, Ningbo 315211, China; jwj1535283070@163.com (W.-J.J.); czhyxn@foxmail.com (Z.-H.C.); 2 Department of Food Science and Engineering, Ningbo University, Ningbo 315211, China; yu2470916005@163.com; 3 College of Electrical and Electronic Engineering, Wenzhou University, Wenzhou 325035, China; 19211710111@stu.wzu.edu.cn

**Keywords:** metal-organic frameworks, fluorescent sensors, hazardous materials

## Abstract

Population growth and industrial development have exacerbated environmental pollution of both land and aquatic environments with toxic and harmful materials. Luminescence-based chemical sensors crafted for specific hazardous substances operate on host-guest interactions, leading to the detection of target molecules down to the nanomolar range. Particularly, the luminescence-based sensors constructed on the basis of metal-organic frameworks (MOFs) are of increasing interest, as they can not only compensate for the shortcomings of traditional detection techniques, but also can provide more sensitive detection for analytes. Recent years have seen MOFs-based fluorescent sensors show outstanding advantages in the field of hazardous substance identification and detection. Here, we critically discuss the application of MOFs for the detection of a broad scope of hazardous substances, including hazardous gases, heavy metal ions, radioactive ions, antibiotics, pesticides, nitro-explosives, and some harmful solvents as well as luminous and sensing mechanisms of MOF-based fluorescent sensors. The outlook and several crucial issues of this area are also discussed, with the expectation that it may help arouse widespread attention on exploring fluorescent MOFs (LMOFs) in potential sensing applications.

## 1. Introduction

Today, excessive emissions of toxic and hazardous substances have become a pressing global issue, as these highly soluble and mobile substances spread easily into the atmosphere, land, and water, resulting in a serious threat to the ecosystem [1]. So far, various luminescence-based chemical sensors have been created for specific hazardous substances detection on the basis of host-guest interactions, resulting in the detection of target molecules down to the nanomolar range [2,3,4,5,6]. Among these luminescence-based probes, sensors based on metal-organic frameworks (MOFs) are more attractive in terms of their structural features, functional composition, and the interactions among MOFs and analytes [7]. The accurate framework of MOFs, in terms of their inherent crystallinity, facilitates a full insight into the sensing behavior of MOFs-analytes during the sensing process. Importantly, the proper selection of the organic and metal compositions provides diverse compositions and porosities enabling simple modulation of MOFs-analyte interactions to facilitate target recognition [8]. LMOFs can be obtained by selecting ligands with fluorescent properties, photoactive metal ions, and modified groups with fluorescence functionalization [9]. For example, the integration of fluorescent linkers bearing aromatic groups or conjugated π-systems and photoactive metal ions into MOFs to generate fluorescent MOFs (LMOFs) can dramatically modulate the luminescence features of MOFs, thus promising different sensing applications [10,11]. Beyond that, modifying groups with fluorescence functionalization and modulating the charge transfer between bridging ligands and metal center ions can also enhance the fluorescence properties of LMOFs. Notably, the pore features of LMOFs (including size, shape, pore environment, etc.) can be engineered and tuned to regulate sensing interactions, which often facilitates sensing performance [12]. 

**Figure 1:**
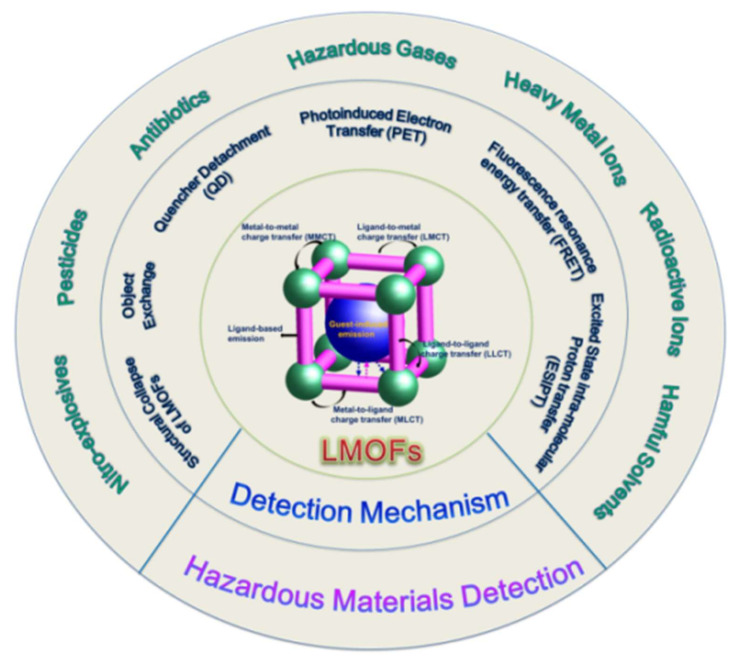
Schematic illustration of emission modes, detection mechanisms, and the applications of MOF-based fluorescent sensors for hazardous materials detection.

In recent years, LMOFs, as one of the more widely used branches of MOFs, have flourished in the fields of optical security displays, biomedical imaging and sensing, and illumination decoration due to their optical tunability and fluorescence diversity, particularly in the field of toxic and hazardous substance detection [13]. Here we highlight the latest advances in sensing applications of MOFs-based fluorescent sensors for hazardous materials, including harmful gases, heavy metal ions, radioactive ions, and a range of harmful organic pollutants, with an emphasis on the influence of composition or structure on the sensing capabilities of LMOFs, as shown in Figure 1. Various luminescence sensing mechanisms and structure-performance relationships are also briefly described. Additionally, the outlooks and several crucial issues of this area are also noted with the expectation of stimulating more attention on investigating the potential of LMOFs for sensing applications.

## 2. Luminous Characteristics of MOF-Based Sensors

The unique pore structure of MOFs restricts the nonradiative leap of organic ligands and promotes the charge transfer between the ligand and the central ion, which results in photoemission and increases the quantum yield and fluorescence efficiency [14]. Therefore, factors like the central metal ion or metal cluster, the structure of the ligand, and the pore size can affect the fluorescence properties of LMOFs. This section will feature an introduction to the luminescence emission patterns and detection mechanism of LMOFs.

### 2.1. Luminous Principle

**Figure 2:**
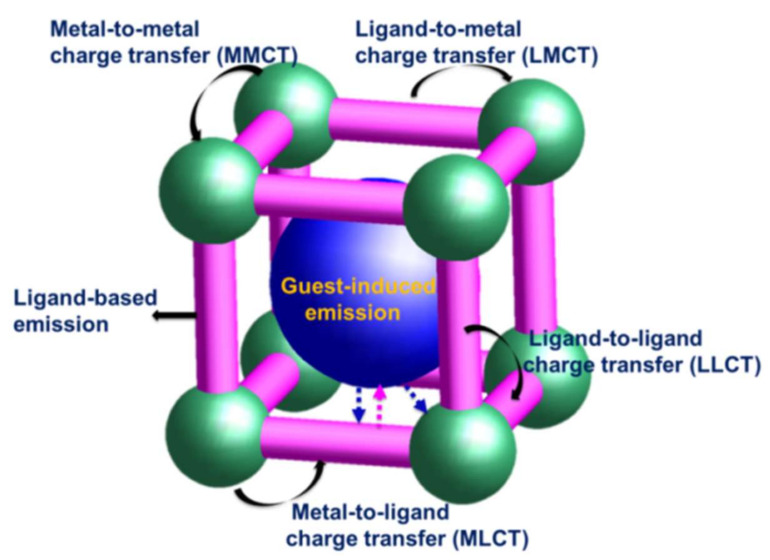
Emission modes of LMOFs.

As a novel platform for the development of traditional photoluminescence materials as well as practical luminescent applications, the fluorescence emission patterns of LMOFs can be classified into the following types, as shown in Figure 2: (1) Emission based on metal ions or metal clusters. LMOFs constructed by lanthanide (Ln^3+^) ions are the most widely used luminescent materials. The f-f electron transition of Ln^3+^ ions is forbidden by Laporte, which makes lanthanide LMOFs have a long fluorescence lifetime and narrow and sharp fluorescence peak shape. All Ln^3+^ ions except La^3+^ and Lu^3+^ can emit significant blue, red, green, orange, and other fluorescent colors. However, due to the forbidden 4f-4f electron leap of lanthanide ions, it becomes particularly difficult to be excited directly, so the sensitization of organic ligands is required, for example the “antenna effect” [15,16,17]. First, the organic linkers of lanthanide MOFs absorb energy under the stimulation of excitation light of certain energy, then the ligands transfer the energy of the excited state to the excitation energy level of Ln^3+^ ions, and finally, the energy of the excited state of Ln^3+^ ions is radiated in the form of photons, emitting characteristic fluorescence based on lanthanide metal ions. Therefore, an organic ligand with a suitable match to the lowest excited state energy level of the rare earth ion must be selected for efficient energy transfer. (2) Emission based on the organic ligand: There are three types of ligand-based luminescent emission, including single ligand emission, intra-ligand charge transfer (ILCT), and inter-ligand charge transfer (LLCT) [18]. In most of the LMOFs reported in the literature, organic ligands with a large number of conjugated structures (e.g., aromatic structures or π-π conjugated systems) can emit energy directly as photons upon excitation by UV or visible light [19]. Single ligand emission and ILCT generally occur in single-ligand MOFs systems, whereas LLCT requires the presence of mixed ligands in the MOFs structure. (3) Charge transfer between the central metal and the organic ligand: The charge transfer is divided into ligand-to-metal charge transfer (LMCT) and metal-to-ligand charge transfer (MLCT), which mainly depends on the relative height of the lowest excited state energy level of the organic ligand and the lowest excited state energy level of the metal ion. If the lowest excited state energy of the organic ligand is lower than the lowest excited state energy of the metal ion, the energy of the metal ion is excited by the outside world and the energy is transferred to the ligand excited state, and the electrons of the ligand excited state then return to the ground state and emit fluorescence, which is the charge transfer from metal to the ligand. Conversely, the charge transfer from the ligand to the metal center occurs. (4) Emission based on the introduced guest molecule: Due to the diverse pore structure and large porosity of MOFs, it is possible to encapsulate the guest chromophores into the pores of MOFs, and the resulting domain-limiting effect can effectively improve the luminescence properties of the system. In addition, it is also possible to incorporate guest molecules into the MOFs surface by post-modification, which is a reliable way to obtain idealized LMOFs.

### 2.2. Detection Mechanism

**Figure 3:**
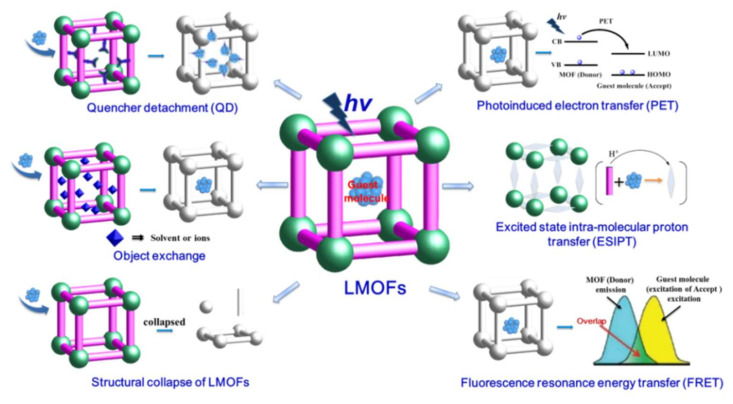
Detection mechanisms of LMOFs.

The adjustable pore size of LMOFs allows them to be used as sieves to selectively allow specific molecules into the pore, and binding sites such as functional groups are abundant on the surface to target specific molecules, all of which can influence their fluorescence emission performance for specific detection of the analyte. LMOFs with large pores can selectively adsorb organic molecules through a domain-limiting effect to achieve detection results [20]. The detection mechanisms of LMOF-based chemosensors could be divided into the following parts, as shown in Figure 3 [21]: (1) Photoinduced electron transfer (PET). Electron transfer between the excited states of the LMOFs (the body) and the analyte (the guest) is induced by light. When the system is excited by light, electrons are transferred from the electron donor to the excited state of the chromophore, at which point the electron donor fluorescence is too weak to be observed. When the analyte binds to the fluorescent probe, the PET process is blocked and the fluorescence emission of the fluorescent chromophore is restored. (2) Excited state intra-molecular proton transfer (ESIPT): When a certain wavelength of excitation light is irradiated to the recognition system, H^+^ ions located in the excited state can be transferred between the proton donor and the acceptor. (3) Fluorescence resonance energy transfer (FRET): A competitive absorption mechanism whereby the excitation or emission energy of a fluorescent probe is absorbed when the absorption spectrum of the analyte overlaps with the excitation or emission spectra of the LMOFs, resulting in luminescence quenching of MOFs. (4) Quencher detachment (QD): Usually, the energy of the C-H bond or -OH vibrations present in organic ligands or solvent molecules is lost through this non-radiative leap, allowing the fluorescence of LMOFs to undergo a burst. When the recognizers are combined with these readily vibrating groups, the energy loss is reduced, allowing the fluorescence to be restored. (5) Object exchange: The fluorescence of MOFs is also burst when the analyte exchanges or binds to a chromophore in the structure of the LMOFs. (6) Structural collapse of LMOFs: During the detection process, the main framework of LMOF collapses due to the presence of the identifier, causing a change in its fluorescence properties and thus causing a fluorescence burst.

## 3. LMOFs for Hazardous Materials Detecting

### 3.1. Hazardous Gases

The rise of global temperature is mainly the result of discharge of harmful gases (CO_2_, SOx, NOx, NH_3_, etc.) into the atmosphere, and these mostly come from human activity, including industry and transportation. These gases can be further shifted to acid/alkaline contaminants, resulting in ozone destruction, photochemical smog, and acid rain [22]. Therefore, a powerful test for these contaminants is required.

Numerous traditional techniques have been adopted for harmful gas detection, like electrochemistry, infrared spectroscopy, GC-MS, and field-effect transistors. Complex instrumentation, large amounts of time, and the need for expensive and bulky equipment limit their widespread use. In addition, some of them cannot tolerate the interference of carbon monoxide and water. Therefore, it is essential to develop dependable harmful gas sensors. Sensors in view of LMOFs have been extensively applied to hazardous gas detection [23]. The hazardous gas detection ability and detection mechanisms of various LMOF-based sensors are summarized in Table 1.

#### 3.1.1. Carbon Dioxide (CO_2_)

CO_2_, as a major greenhouse gas, not only causes global warming and ocean acidification, but also causes symptoms when present above safe CO_2_ levels, such as headache, energy loss, fatigue, inquietude, jelly legs, and pulmonary ventilation problems [24,25]. Molecular systems such as LMOFs are reliable materials that can be applied to CO_2_ sensing [26]. They are made by powerful interactions between metal ions or clusters with easy-to-modify organic linkers and additionally exist with a high surface area and well-defined accessible pores. For example, Qi et al. developed a sensor that relied on the changes in luminescence characteristics of [Zn_7_(ip)_12_](OH)_2_ (ip = 1H-imidazo[4,5-f] [1,10] phenanthroline) [27]. Stability tests show that the framework of [Zn_7_(ip)_12_](OH)_2_ could maintain structural integrity in water, MeOH, or EtOH at room temperature. The photoluminescent property of [Zn_7_(ip)_12_](OH)_2_ is intensely influenced by the existence of CO_2_ molecules. Low-pressure CO_2_ adsorption transfers the discharge color from powerful cyan photoluminescence (λ_max_ = 487 nm) to yellowish orange (λ_max_ = 540 nm). The luminescent mechanisms showed that once loaded guest molecules into [Zn_7_(ip)_12_](OH)_2_, the strain added on the crystal structure of the framework prevents linker-linker interactions, transferring the λ_max_ of its fluorescent discharge.

**Figure 4:**
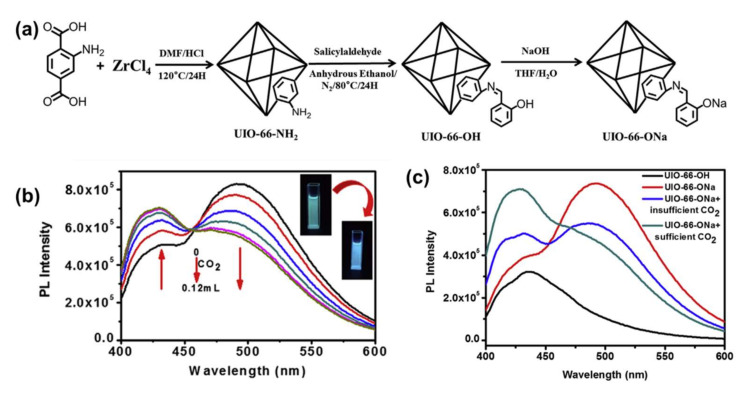
(**a**) The post-synthesis process of UIO-66-ONa. (**b**) Fluorescence spectra of UIO-66-ONa after the gradual bubbling of CO_2_. (**c**) Fluorescence spectra of UIO-66-OH and UIO-66-ONa before and after bubbling of CO_2_. Adapted from [28].

Recently, through the integration of sodium phenolic agents in the framework of UiO-66, Tan et al. obtained a new fluorescent MOF probe, namely UiO-66-ONa, as shown in Figure 4a [28]. The fluorescence lifetimes of UIO-66-NH_2_ were 0.611 (84.41%) and 2.44 ns (15.59%), whereas those of UIO-66-ONa were 1.59 (85.13%) and 6.37 ns (14.87%). UiO-66-ONa shows a very good sensitivity to CO_2_ because of the conversion of the sodium phenol group into an OH group. Once exposed to CO_2_ gas, the fluorescence spectra of UiO-66-ONa and UiO-66-OH are shifted by 50 nm (Figure 4b,c). As a consequence, the concentration of CO_2_ in the surrounding atmosphere could be detected through gauging the fluorescence spectrum of the MOF powder suspension. The detection limit of CO_2_ was as low as 3.5 × 10^−^^7^ M, and the specific reaction between the sodium phenolic group and CO_2_ in solution resulted in its outstanding selectivity over the interference of other ordinary gases, such as CO, N_2_, Ar, and O_2_. The superiority of this material includes excellent chemical selectivity to CO_2_, a well-studied and relatively simple synthesis procedure, and wonderful stability. Stability tests show that the emission intensity did not change significantly after three quenching-recovery cycles, indicating the high reusability of UIO-66-ONa. However, using a suspension is impractical for equipment integration and recycling, requires exposure to NaOH to convert UiO-66-OH to UiO-66-ONa, and indicates that the MOF probe is pH independent, which could limit its efficiency. Further development could conquer the aforementioned limitations, and the concept of a CO_2_ sensor built on a well-known and robust MOF, which can be manufactured by applying the simple synthesis process and has the essential potential for chemical adjustability, is very possible.

#### 3.1.2. Sulphuretted Hydrogen (H_2_S)

H_2_S, which smells like rotten eggs, binds to Fe^2+^ ions in mitochondrial cytochrome enzymes and attacks the central nervous system [29,30]. The U.S. Safety and Health Administration states the permissible exposure to H_2_S should not exceed 10 ppm during an 8 h workday [31,32]. Therefore, the detection of trace levels of H_2_S is of great importance [33,34,35,36]. The original example of the MOF-based fluorescent probe for detecting H_2_S was reported in 2014, namely UiO-66@NH_2_, which suggested an extreme turn-on luminescent response to H_2_S in living cells [37]. After post-modification of UiO-66@NH_2_ using an azidation agent, a highly stable MOF-based sensor UiO-66@N_3_ was obtained. Very weak fluorescent properties in UiO-66@N_3_ were observed because of the existence of an electron-deficient azide agent. However, once UiO-66@N_3_ was treated with Na_2_S in an HEPES (4-(2-hydroxyethyl)-1-piperazineethanesulfonic acid) aqueous buffer (10 mM, pH 7.4), a powerful discharge (16-fold enhancement) was produced, which was attributed to target-mediated azide to amine reduction, resulting in the luminescence of UiO-66@NH_2_. Subsequently, the same group prepared a nitro-functionalised UiO-66@NO_2_ as a fluorescence turn-on probe for H_2_S detection, where UiO-66@NO_2_ can be easily gained in a simple synthetic step by adding 2-nitroterephthalic acid as the organic linker [38]. Recent years have seen the conjunction of azide/nitro moieties with other MOFs, with various luminescent H_2_S sensors being generated, including azide-appended Zn-MOF [39], Ce-MOFs (Ce-UiO-66@N_3_, Ce-UiO-66@NO_2_) [40], Al(OH)(IPA-N_3_) [41], Zr_6_O_4_(OH)_4_((NDC-(NO_2_)_2_)_6_ [42], Al(OH)(BDC-N_3_) [43], and Zr_6_O_4_(OH)_4_(BDC-(NO_2_)_2_)_6_ [44]. All of these functionalized MOF-based probes showed wonderful selectivity and a quick response time for H_2_S.

**Figure 5:**
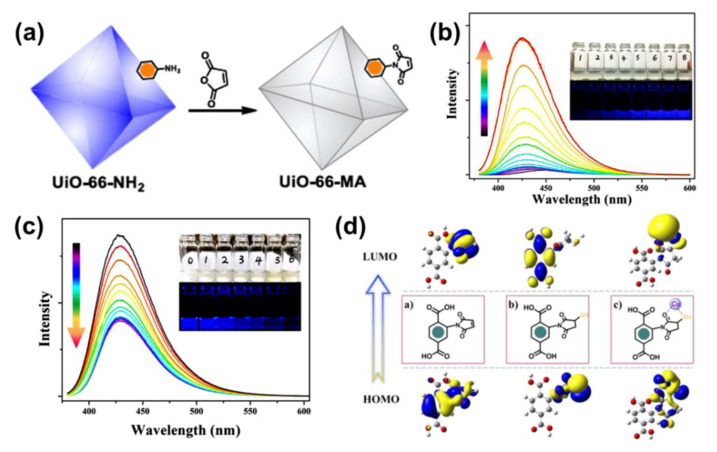
(**a**) The post-synthesis process of UiO-66-MA. (**b**) Fluorescence titration of UiO-66-MA with H_2_S (0-0.625 μM); inset: images of UiO-66-MA under day light (up) and UV light (down, 365 nm) in the presence of 0, 0.04, 0.08, 0.125, 0.165, 0.25, 0.35, and 0.625 μM of H_2_S for 1–8, respectively. (**c**) Fluorescence titration of UiO-66-MA/H_2_S with growing concentration of Cu^2+^; inset: images of UiO-66-MA/H_2_S under day light (up) and UV light (365 nm, down) in the presence of 0, 0.02, 0.07, 0.12, 0.17, 0.22, and 0.35 μM of Cu^2+^ for 0–6, respectively. (**d**) A possible tandem process in view of the Michael addition, and S-Cu integration was calculated by DFT. Adapted from [48].

The binding reaction between S^2−^ and Cu^2+^ can also be adopted for detecting H_2_S [45,46]. For example, the authors of [47] prepared a nano-MOF probe for detecting H_2_S, namely Eu^3+^/Cu^2+^@UiO-66-(COOH)_2_. This MOF-based probe showed two different emissions: 615 nm of Eu^3+^ emission and 393 nm of ligand centered emission. It is noteworthy that H_2_S can enhance and quench the two distinct emissions of the nano-MOF probe, probably ascribed to the affinity between H_2_S and Cu^2+^. Subsequently, the same group created a MOF-based fluorescent logic platform Eu^3+^/Ag^+^@UiO-66-(COOH)_2_ for H_2_S detection [48]. Recently, Yang et al. fabricated a dual functional UiO-MOF sensor by facile post-modification UiO-66-MOF with maleic anhydride (MA), namely UiO-66-MA, as shown in Figure 5a [49]. UiO-66-MA can selectively undergo the Michael addition to H_2_S, accompanied by linear fluorescence turn-on behavior. This MOF-based sensor is extremely selective and sensitive with a detection limit of 3.3 nM, the lowest recorded in all MOF-based H_2_S sensing studies (Figure 5b,c). Mechanistic experiments show that UiO-66-MA exhibits dual sensing function for H_2_S and Cu^2+^ in a tandem process in view of the combined rules about Michael addition and S-Cu integration, as shown in Figure 5d.

#### 3.1.3. Sulfur Dioxide (SO_2_)

**Figure 6:**
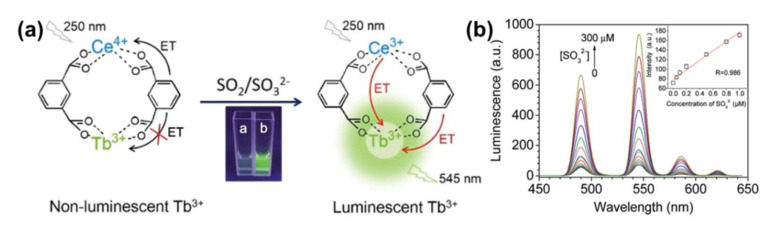
(**a**) Diagram of the Ce^4+^/Tb^3+^ based Ln-MOF for sensing SO_2_ and sulfite via the turn-on of luminescence induced by energy transfer (ET) triggered by redox-reaction. (**b**) The luminescence of Ln-MOF increases with the concentration (0, 0.05, 0.1, 0.2, 0.5, 0.8, 1, 5, 20, 50, 100, 150, 200, and 300 mM) of SO_3_^2−^; the inset shows the linear relationship of the luminescence intensity of Ce-PA-Tb at 545 nm with the concentration of SO_3_^2−^. Adapted from [53].

The excess emission of SO_2_ results in environmental pollution and affects human systems such as the respiratory and nervous systems [50,51]. On study exhibited a sensing paper created from MOF-5-NH_2_ that could selectively identify SO_2_ in various gases (H_2_S, CO_2_, NH_3_, N_2_, NO_2_, and so on) [52]. The detection limit was lower than 0.168 ppm. More significantly, the color change of MOF-5-NH_2_ toward SO_2_ could be monitored with the naked eye, demonstrating a simple method for SO_2_ monitoring. Study [53] designed and synthesized a lanthanide MOF (Ln-MOF) that was made up of a Ce^4+^ ion, a fluorescent Tb^3+^ ion, and m-phthalic acid (PA) for the test of SO_2_ and its derivatives, as shown in Figure 6a. This Ln-MOF based probe is rapidly responsive and steady luminescent for SO_2_ and SO_3_^2−^, and it also suggests high selectivity and wonderful sensitivity, as shown in Figure 6b.

#### 3.1.4. Nitrogen Oxides (NOx)

Nitrogen oxides are polluting gases that can contribute to photochemical smog and acid rain to threaten the environment and biology; they are also significant substances that can affect physiological and pathophysiological processes (e.g., vasodilation, neurotransmission, and immune responses) [54,55,56,57]. By integrating the bright blue emitter of the triphenylamine molecule, two LMOFs of Eu-TCA and Cu-TCA (H_3_TCA = tricarbonyltriphenylamine) were successfully synthesized by [58]. The luminescence of Cu-TCA is enhanced by treatment with 0.1 mM NO, which reduces the Cu^2+^ center to Cu^+^ and can be recovered in aqueous solution. Furthermore, Cu-TCA enables bioimaging applications containing live cells. Additionally, the significant emissions of triphenylamine (430 nm) and lanthanide ion (610 nm) in an Eu-TCA sensor allow it to serve as a ratiometric luminescence probe for NO detection, in pace of the test limit of 140 μM. Study [59] obtained another amine-modified MOF for the detection of NO in aqueous solutions. The addition of 1.0 equivalents of NO reduced the luminescence of UiO-66@NH_2_ by more than 80%, whereas in the presence of 2.0 equivalents of H_2_O_2_, O_2_, OH, ClO^–^, NO^3–^, and NO_2_, the luminescence was not significantly reduced. The results suggest that this amine-modified MOF can be applied as an extremely selective luminescence probe for NO detection.

**Figure 7:**
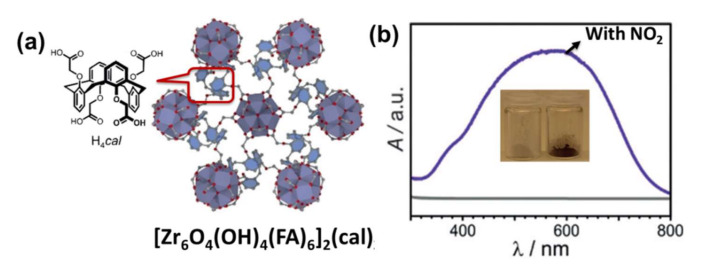
(**a**) The structure of [Zr_6_O_4_(OH)_4_(FA)_6_]_2_(cal)_3_. (**b**) The luminescence intensity changes of [Zr_6_O_4_(OH)_4_(FA)_6_]_2_(cal)_3_ before and after bubbling of NO_2_. Adapted from [60].

As another member of the NOx family, NO_2_ is a hazardous chemical with short- and long-term toxic effects on human health. Schulz et al. characterized a three-dimensional zirconium-based MOF, called [Zr_6_O_4_(OH)_4_(FA)_6_]_2_(cal)_3_ (FA = formate, cal = 1,3-alt-25,26,27,28-tetrakis[(carboxy)methoxy]calixarene), to selectively detect NO_2_ with calixarene linkers, as shown in Figure 7a. [60]. The distinct and reversible color change from white to blue makes the MOF-based probe a visual sensor for detecting of NO_2_ at room temperature, as shown in Figure 7b. Alternatively, two isostructural lanthanide-based MOFs are reported as fluorescent sensors to detect NO_2_ gas [61]. The amino group in the MOFs could act as a sensitive recognition center for NO_2_ molecules. Density functional theory indicated that the shift of energy between the organic linkers and Ln was extremely reliant on the existence of NO_2_, leading to an unprecedented photoluminescent sensing mechanism. Moreover, the authors coated the [Zr_6_O_4_(OH)_4_(FA)_6_]_2_(cal)_3_ on glass slides and investigated them by SEM. Subsequently, they used these coated glass slides for first measurements of NO_2_ in air to evaluate the potential of the novel calixarene-based MOF as a sensor material. The sensing device response to a step function of NO_2_ concentration showed a delay of a few seconds caused by the dead volume of the test setup. The detection of NO_2_ is also possible when humid air is used. No color change was observed when exposing the coatings to NO gas, highlighting the selectivity of the [Zr_6_O_4_(OH)_4_(FA)_6_]_2_(cal)_3_. Heterometallic redox-active MOF in open metal sites and mixed metal nodes may produce specific properties in surfaces and synergistic effects to enhance gas-sensitive performance. For example, Khan et al. obtained a series of hetero-metallic MOFs (Fe_2_^III^M^II^, M = Co, Mn, and Zn) for NO_2_ detection by partially replacing Fe atoms in PCN-250 with the transition metals Co, Mn, and Zn [62]. The morphological and electronic energy band structural properties are controlled by partial metal substitution of Fe while maintaining the PCN-250 framework. The NO_2_ sensing performance at room temperature varies considerably, with Fe_2_Mn PCN-250 showing a greater magnitude of response structure to ppb-level NO_2_ gases compared to pure Fe_3_ PCN-250 and other heterometallic MOFs, mainly due to the supreme binding energy of NO_2_ gases.

#### 3.1.5. Ammonia (NH_3_)

As one of the most widely used industrial gases, NH_3_ is an unstable, corrosive, and colorless gas with an irritating odor. NH_3_ is very toxic, and a minor leak may lead to serious ecological disaster [63]. The National Institute for Occupational Safety and Health (NIOSH) has set a safety alert for NH_3_ exposure: NH_3_ concentrations must not exceed 300 ppm during momentary exposure. Moreover, the U.S. Occupational Safety and Health Administration (OSHA) stipulates that airborne NH_3_ concentrations must not be higher than 50 ppm during long-term exposure due to occupational necessity [64]. 

**Figure 8:**
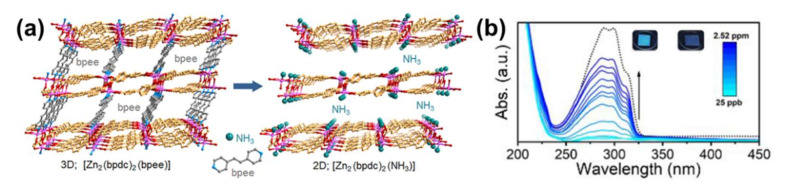
(**a**) The structure of 3D (Zn_2_(bpdc)_2_(bpee) and the schematic representation of the 2D structure potentially obtained after the NH_3_ detection. (**b**) The luminescence intensity changes of (Zn_2_(bpdc)_2_(bpee) immersed in aqueous solutions of NH_3_ with different concentrations. Adapted from [68].

To date, various LMOFs have been carried out to detect trace amounts of NH_3_ [65]. For example, two LMOFs of Mg(H_2_DHBDC) (H_2_DHBDC^2−^ = 2,5-dihydroxybenzene-1,4-dicarboxylate) and Zn_2_(TCPE) (TCPE = tetrakis(4-carboxyphenyl)ethylene) were reported in [66] for NH_3_ detection. The results showed that these two LMOFs could selectively detect NH_3_ at 100 °C. In contrast, their detection of NH_3_ at room temperature did not exhibit selectivity, indicating that temperature is a significant parameter for luminescence sensing. Although the mechanism of the above consequences is still unknown, this task provides a new way to gain luminescent tests with high selectivity. Additionally, study [67] also highlighted a turn-on luminescent test for NH_3_ applying LMOFs structured by Eu^3$#x2212;^ functionalization MOF and exchange of linker, respectively. Significantly, all of them have the ability to test for NH_3_ in biological systems. Recently, ref. [68] obtained a LMOF (Zn_2_(bpdc)_2_(bpee), H_2_bpdc = 4,4′-biphenyldicarboxylic acid, bpee = 1,2-bipyridylethene) containing mixed matrix membranes (MMMs) by a simple solvent-free method to detect trace amounts of amines in gas phase. The structure of (Zn_2_(bpdc)_2_(bpee) and sensing mechanism are shown in Figure 8a. After exposing the LMOF-containing MMMs to amines, the bpee exchange by the amine occurred, triggering an absorption and luminescence response ascribed to the release of bpee molecules inside the LMOF. With the increase of NH_3_ content from 280 ppb to 28 ppm, the absorption opening is consistent with the gradual evolution of the PL spectrum from broad band with a maximum of 459 nm to narrow PL with a peak of 403 nm, as shown in Figure 8b.

**Table 1:** The gas detection ability and detection mechanisms of various LMOF-based sensors.

Analyte	MOF	Detection Limit	Water Stability	Detection Mechanism	Ref
CO_2_	[Zn_7_(ip)_12_](OH)_2_	-	High	QD	[27]
	UiO-66-ONa	3.5 × 10^−7^ M	High	QD	[28]
H_2_S	UiO-66@NH_2_	118 μM	High	Target-mediated azide to amine reduction	[37]
	UiO-66@NO_2_	188 μM	High	Target-mediated nitro group to amine reduction	[38]
	Zn-MOF	28.3 μM	High	Target-mediated nitro group to amine reduction	[39]
	Azide functionalized Ce-MOFs Nitro functionalized Ce-MOFs	12.2 μM 34.8 μM	High	Target-mediated azide/nitro groups to amine reduction	[40]
	Al(OH)(IPA-N_3_)	2.65 μM	High	Target-mediated N_3_ to amine reduction	[41]
	Zr_6_O_4_(OH)_4_((NDC-(NO_2_)_2_)_6_	20 μM	High	Target-mediated nitro group to amine reduction	[42]
	Al(OH)(BDC-N_3_)	90.47 nM	High	Target-mediated N_3_ to amine reduction	[43]
	Zr_6_O_4_(OH)_4_(BDC- (NO_2_)_2_)_6_	14.14 μM	High	Target-mediated nitro group to amine reduction	[44]
	Eu^3+^/Cu^2+^@UiO-66- (COOH)_2_	-	High	Quench the broad LC emission through its superior affinity for Cu^2+^ ions	[47]
	Eu^3+^/Ag^+^@UiO-66- (COOH)_2_	23.53 μM	High	-	[48]
	UiO-66-MA	3.3 nM	High	Michael addition to H_2_S	[49]
SO_2_	MOF-5-NH_2_	0.168 ppm	High	-	[52]
NO	Cu-TCA Eu-TCA	0.1 mM 140 μM	High	Cu^2+^ ions quench the ligand-based fluorescence Formation of coordination bonds between the europium and NO	[58]
	UiO-66@NH_2_	0.575 μM	High	-	[59]
NO_2_	{[Tb_2_(NBDC)_3_(DMF)_4_]·2DMF} {[Eu_2_(NBDC)_3_(DMF)_4_]·2DMF}	1.8 ppm 2.2 ppm	High	QD	[61]
	Fe_2_^III^M^II^, M = Co, Mn, and Zn	500 ppb	High	QD	[62]
NH_3_	Mg(H_2_DHBDC) Zn_2_(TCPE)	-	High	FRET	[66]
	Eu^3^-functionalization MOF	2.4 ppm	High	PET	[67]
	(Zn_2_(bpdc)_2_(bpee)	50 ppm	High	FRET	[68]

### 3.2. Hazardous Metal Ions

#### 3.2.1. Heavy Metal Ions

There are three representative environmental pollutants caused by industrial effluents: lead (Pb), mercury (Hg), and cadmium (Cd) [69]. Even when detected in minute quantities, they can do deadly damage to biological systems. To date, several facile and novel analytical methods for the detection of heavy metal ions have been established, including atomic absorption spectroscopy, cold vapor atomic fluorescence spectrometry, inductively coupled plasma mass spectrometry, electrochemical methods, high performance liquid chromatography, and gas chromatography. However, expensive equipment and complicated sample preparation processes limited their widespread use. Thus detection technology with high precision at the ppb level is critical for environmental monitoring, yet remains a challenge. Fluorescent MOF-based sensing is widely used in the field of chemical analysis and detection due to its high sensitivity, quick response, and easy operation. The heavy metal ion detection ability and detection mechanisms of various LMOF-based sensors are summarized in Entry 1 of Table 2.

**Figure 9:**
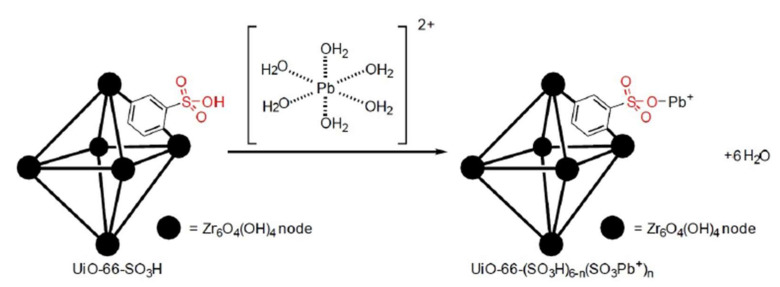
Diagram of adsorption of [Pb(OH_2_)_6_]^2+^ on SO_3_H-UiO-66(Zr) in water. Adapted from [74].

Lead Ion (Pb^2+^): Pb^2+^, as the leading heavy pollutant ion, can produce fatal effects to human health and lead to a series of incurable diseases. Therefore, it is of great significance to explore a quick and precise method to detect Pb^2+^ ions. A millimeter level lanthanide-based MOF sensor was synthesized by Ji et al. for detecting the Pb^2+^ ion, namely [Tb(L)(H_2_O)_5_]*_n_* (H_2_L = 3,5-dicarboxyphenol) [70]. Tb-MOF demonstrated a quite sensitive green luminescence provoked by the efficient antenna effect of the ligands, and the detection limit for Pb^2+^ ions was as low as 10^−^^7^ M. The reason why Tb-MOF became a trustworthy and simple-to-operate luminescent sensor and is used for pollutant detection is the ideal line type relation between the luminescence intensity of the crystal and the concentration of the Pb^2+^ ions. Subsequently, the authors of [71] fabricated three Cd(II) iso-frameworks to detect a trace quantity of Pb^2+^ by reacting the BIPA ligand (BIPA = bis(4-(1*H*-imidazol-1-yl)phenyl)amine) and different carboxylic ligands (H_2_IPA = isophthalic acid, H_2_HIPA = 5-hydroxyisophthalic acid, H_2_NIPA = 5-nitroisophthalic acid) with Cd(II), namely {[Cd(BIPA)(IPA)]·DMF}*_n_*, {[Cd(BIPA)(HIPA)]·DMF}*_n_*, and {[Cd(BIPA)(NIPA)]·2H_2_O}*_n_*. The fluorescence detection experiments showed that {[Cd(BIPA)(IPA)]·DMF}*_n_* and {[Cd(BIPA)(HIPA)]·DMF}*_n_* are dual-reactive photoluminescent sensors for Hg^2+^ and Pb^2+^ ions with low detection concentration and high quenching constant. Wang et al. tested two kind of luminescence-active transition MOFs, Zn-MOF and Cd-MOF, and carried out the sensing experiments [72]. The detection results prove that even with variable frameworks, both of these two transition MOFs present multiple-target detection for Pb^2+^ with high sensitivity, fair anti-interference capacity, and great recyclability. Competitive energy absorption between MOFs and the analytes made a contribution to the recognition mechanism. Aggregation-induced emission (AIE) in fluorescent probe can also be adopted to detect metal ions in aqueous solutions. For example, by reacting with zinc (Zn^2+^) ions, H_2_TCPP and TPE-2COOH, zinc porphyrin-based MOF (Zn-TCPP-MOF) with AIE was successfully obtained by Wang et al. [73]. Excellent fluorescent property of Zn-TCPP-MOF allows it to be used as a fluorescent probe to detect Pb^2+^ ions in aqueous solution. In this experiment, the Zn-TCPP-MOF with a bright red color converted to being colorless after the mixture of Pb^2+^ ions, which can be observed without any devices. At the same time, when the content of Pb^2+^ ions is at quite a low level, a perfect linear relationship between fluorescence quenching efficiency and concentration was illustrated. When compared with other MOFs, the Zn-TCPP-MOF detected Pb^2+^ ions in water, at very low concentrations, as well as at its fully lower limit of detection (LOD) of 4.99 × 10^−^^8^ M. The SO_3_H-UiO-66(Zr) MOF system, functionalized with an SO_3_H group and usually installed in the end-face of an optical fiber, implemented the function of detecting Pb^2+^ in water at 25.2, 43.5, and 64.0 ppm levels [74]. The proposed removal mechanism is based on the adsorption of [Pb(OH_2_)_6_]_2+_ in water on SO_3_H-UiO66(Zr) because of a strong affinity between functionalized MOF and lead, as shown in Figure 9.

Mercury Ion (Hg^2+^): The mercury ion Hg^2+^, a highly toxic heavy metal, can be converted to methylmercury, a powerful neurotoxin that can accumulate through the food chain and be ingested by humans to cause serious symptoms. These matters have contributed significantly to the rapid revolution in Hg^2+^ ion detection technology [75,76,77].

**Figure 10:**
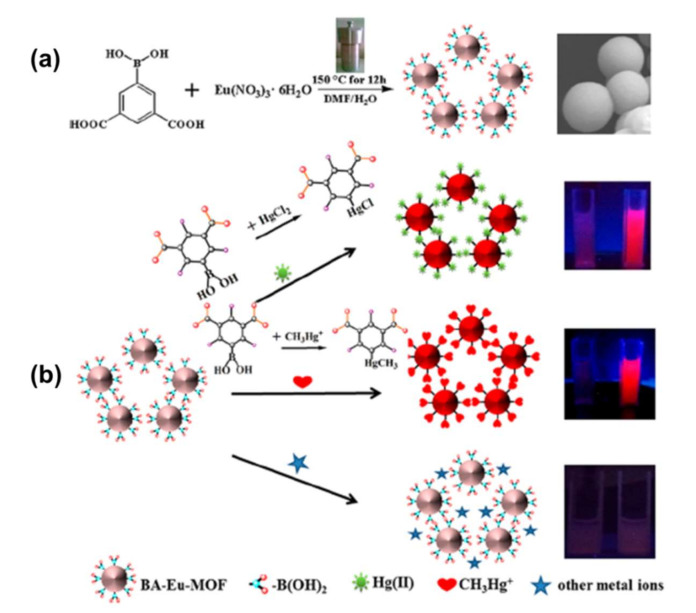
(**a**) Synthesis process of BA-Eu-MOF. (**b**) Schematic diagram of the sensing process of BA-Eu-MOF toward Hg^2+^ and CH_3_Hg^+^ ions based on transmetalation. Adapted from [82].

Based on the modifiable host-guest interactions among LMOFs and analytes, many MOFs with nitrogen centers, amine groups, and alkyne groups have been engineered to detect Hg^2+^ [78]. For example, ref. [79] fabricated a butyne-modified luminescent MOF probe with high chemical and hydrolytic stability in water, namely UiO-66@butyne, to detect Hg^2+^ through the oxymercuration process. UiO-66@butyne was obtained by the reaction of a Zr ion and 2,5-bis (but-3-yn-1-yloxy) terephthalic acid, in which the butyne moiety acted as the recognition site for Hg^2+^. The MOF also showed high selectivity for Hg^2+^ with a significant LOD of 10.9 nmol L^−1^ and a rapid response time of 3 min. Zhang et al. obtained a series of amino-functionalized LMOFs with high oxidation state central metals (Al^3+^, Zr^4+^, Cr^3+^, Fe^3+^, and Ti^4+^), enabling trace detection of Hg^2+^ through strong coordination of amino groups with Hg^2+^ [80]. Additionally, the intrinsic fluorescence intensity of MOFs is dominated by the effect of LMCT. Particularly, NH_2_-MIL-53(Al) demonstrated an outstanding ability for Hg^2+^ detection with a wide response range (1–17.3 μM), quite low detection limit (0.15 μM), excellent selectivity, broader pH adaptation (4.0–10.0), and strong anti-interference capacity. Khatun et al. produced a thiazolothiazole-based LMOF with Hg^2+^-sensing capabilities, namely Zn_2_(NDC)_2_(DPTTZ), in which naphthalene dicarboxylate (NDC) struts served as antenna chromophores and energy donors and *N*,*N′*-di(4-pyridyl)thiazolo-[5,4-*d*]thiazole (DPTTZ) pillars as complementary energy acceptors and light emitters [81]. In the presence of Hg^2+^, the photoluminescence of Zn_2_(NDC)_2_(DPTTZ) MOF underwent a significant red-shift to 450 nm followed by quenching. The average fluorescence lifetimes of this material in the presences of different concentrations of Hg^2+^ were calculated as 9.57, 7.89, and 7.25 ns, respectively. Recently, a fluorescence “turn-on” probe (BA-Eu-MOF) with boric acid (BA) containing ligand 5-boronobezene-1,3-dicarboxylic acid (5-bop) was prepared to detect Hg^2+^ and CH_3_Hg^+^ by Wang et al., as shown in Figure 10a [82]. The BA-Eu-MOF demonstrated a feeble red emission in water due to the passivating “antenna” effect of the ligand caused by the electron withdrawing phenomenon of the BA moiety. A transmetalation reaction (Hg^2+^ or CH_3_Hg replaced the BA agent) took place based on the effects described above, as shown in Figure 10b. In this way, the “antenna” effect of the ligand was touched, resulting in the improvement of red emission. With the red emission increased for improved concentration of Hg^2+^ and CH_3_Hg, the chromatism can also be seen by the naked eye under 365 nm ultraviolet light. Because of the porous property of the MOF and its inherent surface effect, along with the particular transmetalation reaction between the BA agent and Hg^2+^ or CH_3_Hg, the well optimized nano-probe presented some excellent characteristics for simultaneous Hg^2+^ and CH_3_Hg detection, such as brief preparation, easy operation, “turn-on” signal output, great selectivity, and high sensitivity. Helal et al. obtained a fluorescein hydrazide-appended Ni(MOF) [Ni_3_(BTC)_2_(H_2_O)_3_]·(DMF)_3_(H_2_O)_3_ for probing Hg^2+^ ions [83]. It has been proven that this composite, in the state of aqueous emulsion, could generate a new peak in absorption at 583 nm, with a chromogenic change to pink observed by the naked eye after a mixture with Hg ions. Moreover, this compound enhances its fluorescence with a chromatic transform to green fluorescence upon hybridization with the Hg ions. The binding constant was found to be 9.4 × 10^5^ M^−1^, with a detection restriction of 0.02 μM or 5 ppb. The sensor also demonstrated that it is reversible and could be used seven times. Moreover, the detection of Hg^2+^ ions in water samples of groundwater, tap water, and drinking water was also tested.

**Figure 11:**
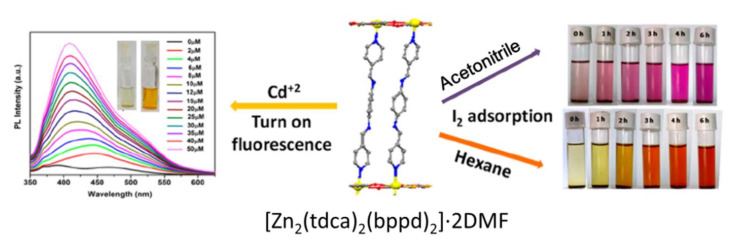
Structure of [Zn_2_(tdca)_2_(bppd)_2_]·2DMF, UV-vis spectra of [Zn_2_(tdca)_2_(bppd)_2_]·2DMF in acetonitrile suspension upon addition of Cd^2+^ at room temperature (λ_ex_ = 330 nm), and photographs of the iodine release process in acetonitrile and hexane. Adapted from [86].

Cadmium Ion (Cd^2+^): Cd^2+^ is extremely poisonous to both the environment and human physiology. Long-term inhalation of cadmium can produce chronic poisoning and cause kidney damage. So far, much attention has been paid on the use of LMOFs for probing Cd^2+^. By incorporated perovskite quantum dots (CH_3_NH_3_PbBr_3_) into MOF-5, study [84] formed a compound of CH_3_NH_3_PbBr_3_@MOF-5 that exhibited good moisture resistance properties even after direct exposure to water for 30 days and could sense Cd^2+^ by enhanced luminescence. By utilizing the pyrrole Lewis base site as a luminescent moiety, Moradi et al. obtained a porphyrinic zirconium-based LMOF for highly selective sensing of Cd^2+^, in which Cd^2+^ can interact with pyrrole Lewis base site, resulting in a luminescent quenching [85]. Recently, Mandal et al. synthesized a porous MOF-based probe [Zn_2_(tdca)_2_(bppd)_2_]·2DMF by using the ligand thiophene-2,5-dicarboxylic acid (H_2_tdca), co-ligand N,N′-bis(4-pyridylmethylene)-1,4-benzenediamine (bppd), and Zn(NO_3_)_2_. It was found that [Zn_2_(tdca)_2_(bppd)_2_]·2DMF is an excellent fluorescence probe for the detection of toxic Cd^2+^ ion selectivity and sensitivity, as shown in Figure 11 [86]. Although an increasing number of LMOF-based sensors have been reported as possible probes for the detection of Cd^2+^ ions, these were mostly employed in DMFs, restricted by their poor water stability. Further explorations are needed to detect Cd^2+^ in water [87].

#### 3.2.2. Radioactive Ions

Trace amounts of nuclear waste-related metal ions (UO_2_^2+^, Th^4+^, TcO_4_^−^, and ReO_4_^−^) detected in the environment is a thorny issue due to their detrimental influence on human health and the environment [88]. Nuclides are usually present in the form of anions and cations. The current treatment methods for radioactive ion detection can be generally classified into physical methods, chemical methods, and other methods, specifically including co-precipitation, ion exchange, adsorption, electrocoagulation, solvent extraction, redox, biological methods, and membrane separation technology. Compared with other technologies, fluorescence detection technology, especially fluorescent MOF-based sensing, has more advantages, such as simple equipment setup, easy operation, high safety, high efficiency, low consumption, good regeneration performance, and less harmful secondary products. Recently, LMOFs with good chemical stability and abundant functional groups have been developed to effectively detect these radioactive ions in an aqueous environment. The radioactive ion detection ability and detection mechanisms of various LMOF-based sensors are summarized in Entry 2 of Table 2.

**Figure 12:**
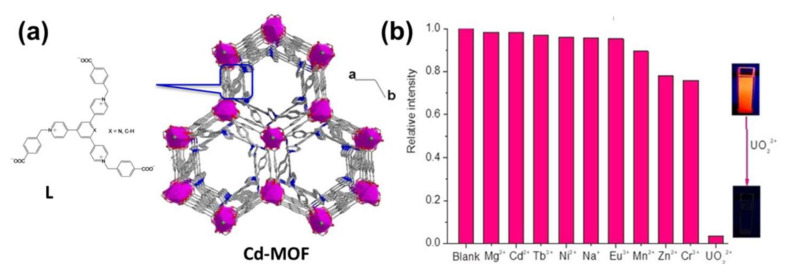
(**a**) Structure of L and Cd-MOF. (**b**) Fluorescence response of Cd-MOF to different metal ions. Adapted from [94].

Uranium Ion (UO_2_^2+^): UO_2_^2+^ has been extensively used in the development of nuclear technology, and there have been some unpredictable leaks over the last few decades. Very trace amounts of UO_2_^2+^ in a human being can cause cancer and acute kidney and liver damage. Therefore, it is important to explore a new approach with high selectivity, high detection sensitivity, and operational simplicity to detect uranium. Liu et al. reported on a Tb-MOF with large channels to accommodate UO_2_^2+^. Using the Tb-MOF, a luminescent detection of UO_2_^2+^ can be realized in water and the LOD is 0.9 μg L^−1^ [89]. Compared with the above study, the LOD limit in water demonstrates a better practical application. Subsequently, they synthesized another Ln-MOF based sensor for detecting UO_2_^2+^, namely [Eu_2_(MTBC)(OH)_2_(DMF)_3_(H_2_O)_4_]·2DMF·7H_2_O (MTBC = 4′,4′,4′,4′-methanetetrayltetrakis-[1,1′-biphenyl]-4-carboxylic acid) [90]. The detection limit towards UO_2_^2+^ ions is 309.2 μg/L. Recently, many La-based LMOFs (CH_3_)_2_NH_2_[Ln_2_(BTC)(AC)_3_(FM)], (Ln = Pr, Ce, Nd, Eu, Sm, Gd, Tb, Ho, Er, and Yb, H_3_BTC = 1,3,5-benzenetricarboxylic acid) were obtained by adding H_3_BTC and Ln(NO_3_)_3_·6H_2_O with mixed formic acid (FM) into glacial acetic acid (AC) [91]. The authors indicated that (CH_3_)_2_NH_2_[Eu_2_(BTC)(AC)_3_(FM)] had rapid detection capabilities for UO_2_^2+^ in an aqueous solution with a lower detection limit of 4.12 μM. At the same time, the quenching rate for UO_2_^2+^ can reach 98.01%. In addition to La-based LMOFs, a carboxyl-functionalized 3D Zn-based LMOF [Zn(HL)(bipy)_0.5_(H_2_O)]·2H_2_O ([H_3_L = 9-(4-carboxy-phenyl)-9*H*-carbazoly-3,6-dicarboxylic acid, bipy = 4,4′-bipyridine]) was fabricated by Hou et al. with sensing ability for UO_2_^2+^ ions in an aqueous solution by fluorescence quenching [92]. Chen and Wang obtained a series of 2D isomorphous MOFs [M(HBTC)(BMIOPE)·DMF·H_2_O]_n_ (M = Zn, Zn_0.7_Co_0.3_, Zn_0.5_Co_0.5_, Zn_0.3_Co_0.7_, Co, BMIOPE = 4,4′-bis(2-methylimidazol-1-yl)diphenyl ether, H_3_BTC = 1,3,5-benzenetricarboxylic acid) to discuss the optical detection of UO_2_^2+^ in an aqueous solution [93]. Seven isostructural MOFs indicating novel 2D→2D supramolecular entanglement featuring catenane-like interlocking of tricyclic cages by the assembly of two tripyridinium-tricarboxylate ligands and different metal ions were reported by Guo et al., as shown in Figure 12a [94]. The tripyridinium-afforded and metal-modulated photoresponsive properties of Cd-MOF allow it to be used as a potential fluorescence sensor for sensitive and selective detection of UO_2_^2+^ in water, as shown in Figure 12b. The photoresponse in both light absorption (color) and emission has the appeal for applications in dual-output optical devices. Mechanistic experiments showed that network entanglement dictates close donor-acceptor contacts, which make fluorescence originating from ILCT. These contacts also allow photo-induced electron transfer, which is the basis for the photochromic and corresponding fluorescence reactions. Metal dependence in fluorescence and photochromism can be bound up with energy transfer through metal-centered d-d conversions. Importantly, LMOF-based sensors are highly capable of detecting UO_2_^2+^ ion in natural water systems such as lake and sea water, which provides an excellent development strategy for UO_2_^2+^ detection.

**Table 2:** The hazardous metal ion detection ability and detection mechanisms of various LMOF-based sensors.

Entries	Analyte	MOF	Detection Limit	Detection Mechanism	Refs
Entry 1	Pb^2+^	[Tb(L)(H_2_O)_5_]*_n_*	10^−^^7^ M	QD	[70]
		{[Cd(BIPA)(IPA)]·DMF}*_n_*	7.5 × 10^−^^7^ M	QD	[71]
		{[Cd(BIPA)(HIPA)]·DMF}*_n_*	5.0 × 10^−^^7^ M		
		Zn-TCPP-MOF	4.99 × 10^−^^8^ M	-	[73]
		SO_3_H-UiO-66(Zr)	25.2 ppm	QD	[74]
	Hg^2+^	UiO-66@butyne	10.9 nmol/L	QD	[79]
		NH_2_-MIL-53(Al)	0.15 μM	QD	[80]
		Zn_2_(NDC)_2_(DPTTZ)	-	FRET	[81]
		[Ni_3_(BTC)_2_(H_2_O)_3_]·(DMF)_3_(H_2_O)_3_	0.02 μM	QD	[83]
	Cd^2+^	Zr-based LMOF	0.002 μM	PET	[85]
		[Zn_2_(tdca)_2_(bppd)_2_]·2DMF	0.132 μM	PET	[86]
Entry 2	UO_2_^2+^	Tb-MOF	0.9 μg/L	PET	[89]
		[Eu_2_(MTBC)(OH)_2_(DMF)_3_(H_2_O)_4_]·2DMF· 7H_2_O	309.2 μg/L	PET	[90]
		(CH_3_)_2_NH_2_[Ln_2_(BTC)(AC)_3_(FM)]	4.12 μM	QD	[91]
		[M(HBTC)(BMIOPE)·DMF·H_2_O]_n_	2.47 × 10^−^^5^ M	FRET	[93]
		Cd-MOF	8.5 μg/L	PET	[94]
	Th^4+^	[Eu_2_(FDC)_3_(DMA)_2_]·4H_2_O	3.49 × 10^−^^5^ mol/L	PET FRET	[95]
		[Ln_2_(NH_2_-BDC)_2.5_(CH_3_COO)(DMA)(H_2_O)]·DMA	3.40 μM	QD	[96]
	^99^TcO^4−^	[1H_6_(ReO_4_)](CF_3_-SO_3_)_5_·7H_2_O	-	PET	[97]
		Cd(II)-MOFs	0.68 × 10^4^ M^−1^	-	[98]
	ReO_4_^−^	[Zr_6_O_4_(OH)_4_(NH_3_^+^-BDC)_6_]Cl_6_·*x*H_2_O (MOR-1)	0.36 ppm	PET	[99]
		H_16_[Zr_6_O_16_(H_2_PATP)_4_]Cl_8_·*x*H_2_O (MOR-2)	0.20 ppm		
		[Ag(1,2,4,5-p4b)](SbF6) (TJNU-302)	-	FRET ESIPT	[100]

**Figure 13:**
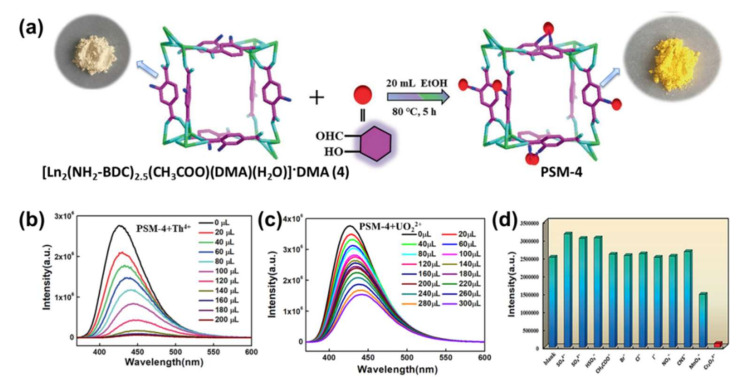
(**a**) Synthetic route of the post-synthesis modified PSM-4. (**b**) Fluorescence spectra of PSM-4 for the detection of Th^4+^. (**c**) Fluorescence spectra of PSM-4 for the detection of UO_2_^2+^. (**d**) Fluorescence intensity of PSM-4 with the addition of different anions. Adapted from [96].

Thorium Ion (Th^4+^): The thorium ion Th^4+^ is a radioactive contaminant and can give rise to some incurable diseases, such as bone and lung cancers. It is essential for both nuclear science and environmental protection to explore a precise and quick way to detect thorium ions. However, only two examples of LMOF-based probes for Th^4+^ ion detecting have so far been published. In 2019, the authors of [95] designed a 3D MOF, [Eu_2_(FDC)_3_(DMA)_2_]·4H_2_O, with excellent hydrolytic stability by using 2,5-furan dicarboxylic acid (2,5-H_2_FDC) as an organic linker. [Eu_2_(FDC)_3_(DMA)_2_]·4H_2_O presented excellent sensing ability toward the Th^4+^ ion in water with the ^5^D_0_ lifetime as long as 1.087 ms. The experimental detection limit of 3.49 × 10^−^^5^ mol L^−1^ was realized. Additionally, 80–95% of Th^4+^ ions were recognized by this Eu-MOF with a mixture of other metals. The sensing mechanism indicated that the competitive absorption of the excitation light significantly contributed to the luminescence quenching, whereas the high uptake and sensing capacity toward Th^4+^ were mainly caused by host-guest interaction. In 2021, Li et al. proposed a series of 3D LMOFs, [Ln_2_(NH_2_-BDC)_2.5_(CH_3_COO)(DMA)(H_2_O)]·DMA, Ln = Pr, Nd, Sm, Eu, Gd, and Tb, NH_2_-BDC = 2-aminoterephthalic acid, DMA = *N,N*-Dimethylacetamide) by hydrothermal synthesis, as shown in Figure 13a [96]. A series of post-synthesis modified materials with great fluorescence and quite stable structure were finally synthesized by an aldimine reaction, due to the existence of uncoordinated amino groups in the organic ligands. These post-synthesis modified materials were capable of being used as potential fluorescence sensors for Th^4+^, UO_2_^2+^, and Cr_2_O_7_^2−^ detection, and the calculated result proved that these modified probes are more sensitive to the detection of analytes (Th^4+^, UO_2_^2+^, and Cr_2_O_7_^2−^) than coordination polymers, as shown in Figure 13b–d. Moreover, the fluorescence lifetime of post-synthesis modified material was 1.39 ms; when analytes was added, the fluorescence lifetimes were 1.33 ms (Th^4+^), 1.36 ms (UO_2_^2+^), and 1.33 ms (Cr_2_O_7_^2−^), respectively.

Pertechnetate Ion (TcO_4_^−^): The ion ^99^TcO^4−^ has a long half-life period and high radioactivity, and it is not only a potential radiation hazard, but also has redox activity and high environmental mobility. Therefore, the selective and efficient detection of ^99^TcO^4−^ has been an urgent issue in the field of environmental radiochemistry. However, very few LMOFs have been discovered for detecting TcO^4−^. The first LMOF-based sensor for TcO^4−^ detection in water was proposed by Amendola et al. in 2014 [97]. Subsequently, a study utilized tris(4-imidazolylphenyl)amine (TIPA) to synthesize a series of Cd(II)-MOFs with pores occupied by different anions [98]. The experimental results of Cd(II)-MOFs proved that only the Cd(II)-MOF with ClO^4−^ can be quenched by ^99^TcO^4−^, along with a quenching constant value of 0.68 × 104 m^−1^. The strong interaction and similar sorption wavelength between Cd(II)-MOFs and guest anions may be responsible for the presented phenomenon.

**Figure 14:**
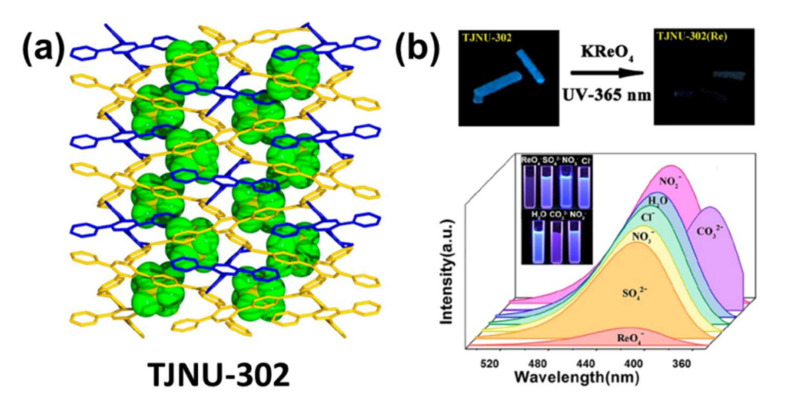
(**a**) Structure of TJNU-302. (**b**) UV photos of TJNU-302 crystals before and after immersion in KReO_4_ water solution and emission spectra of TJNU-302 in water solutions containing different anions. Adapted from [100].

Perrhenate Ion (ReO_4_^−^): One of the most difficult anions to handle in nuclear contaminated waste, ReO_4_^−^ is almost identical in structure, charge density, size, and physicochemical properties with ^99^TcO^4−^. The first example of using LMOF-based probes to detect the ReO_4_^−^ ion was reported by Rapti et al. They reported two Zr-based MOFs [Zr_6_O_4_(OH)_4_(NH_3_^+^-BDC)_6_]Cl_6_·*x*H_2_O (MOR-1) and H_16_[Zr_6_O_16_(H_2_PATP)_4_]Cl_8_·*x*H_2_O (MOR-2) to achieve adsorption and detection of ReO_4_^−^ and TcO_4_^−^ [99]. MOR-1 and MOR-2 showed great absorbability for ReO_4_^−^ and TcO_4_^−^ anions. Importantly, both of these two compounds exhibit selective luminescence sensing properties for ReO_4_^−^. An additional study explored a cationic framework of [Ag(1,2,4,5-p4b)](SbF6) (TJNU-302), exhibiting an apparent emission peak at 416 nm under excitation at 324 nm, as shown in Figure 14a,b [100]. After the mixture of ReO^4−^, a relative high quenching percentage of almost 86% was achieved. This compound also obtained the highest detection limit value of 90 μm compared to other reported ReO^4−^ sensors. Moreover, the sensing behavior of the TJNU-302 is independent of the pH of the solution, further indicating its hydrolytic stability. Even in the simulated Hanford Roche melter circulation stream, quenching percentages of 4.6–27.7% were still observed, indicating that TJNU-302 has good practical applications as a luminescence sensor for ReO^4−^.

### 3.3. Hazardous Organic Pollutants

#### 3.3.1. Antibiotics

Antibiotics, including aminoglycosides, beta-lactams macrolides, amino alcohols, tetracyclines (TC), lincosamide peptides, antifungals, and antineoplastics, are drugs with immunosuppressive effects. Abuse of antibiotics can lead to super antibiotic resistance. In addition, antibiotics can deteriorate and cause organic pollution, which does not naturally degrade. As the concentration of antibiotics in wastewater is relatively low, the detection of antibiotics is usually done with trace analysis, often using instruments with high sensitivity. The main techniques used by various research institutes for the detection of antibiotics in livestock wastewater are chromatography and its coupling techniques, enzyme immunoassay, and capillary electrophoresis. However, all these methods are time consuming, expensive, and require complex equipment and trained personnel. Alternately, optical sensing and adsorption-based methods have been considered as promising technologies in the detection and removal of antibiotics and organic explosives, respectively, because of some advantages such as easy operation, low energy use, high efficiency, and so on.

Detection technology of antibiotic based on LMOFs was initially explored in 2016 [101]. The LOD for nitrofurazone (NZF) and 2,4,6-trinitrophenol (TNP) could be up to 58 and 90 ppb, respectively, when used with the sensors based on this Zr(IV)-MOF. Enlightened by this method, an increasing number of LMOFs have been shown to be useful for the detection of antibiotics, particularly TCs and nitrofurans (NFs). The antibiotics detection ability and detection mechanisms of various LMOF-based sensors are summarized in Entry 1 of Table 3. The authors of the article had also synthesized a series of Cd(II)-based LMOFs to detect antibiotics, namely [Cd_2_Na(L)(BDC)_2.5_]·9H_2_O, [Cd_2_(L)(2,6-NDC)_2_]·DMF·5H_2_O and [Cd_2_(L)(BPDC)_2_]·DMF·9H_2_O (L = *N*^1^-(4-(1H-1,2,4-triazole-1-yl)benzyl)-*N*^1^-(2-aminoethyl)ethane-1,2-diamine) [102]. The LODs of these MOFs sensors for NZF detection are approximately 162, 75, and 60 ppb, respectively. Both fast reaction characteristics and high sensitivity for trace amounts of antibiotic were demonstrated by the experimental results of these MOFs. Since then, a series of chemical sensors based on Cd-MOFs have been reported for the detection of explicit antibiotics. For example, a PET-based photochromism 2D Cd-based LMOF was obtained from a tris(pyridinium)-based hexacarboxylate zwitterionic organic linker [103]. The 2-fold 2D→2D parallel entanglement structure determined the tight interlayer contact between the carboxylate (electron donor) and pyridinium (acceptor), which in turn gives the MOF its reversible photochromic properties. In addition, the fluorescence property of Cd-LMOF in water dispersion is highly selective for nitrofuran antibiotics with high selectivity. Recently, various Cd-containing LMOFs sensors were generated, including {[Cd(HL)(bic)(H_2_O)]·2H_2_O}, {[Cd_3_(L)_2_(bbi)_2_(H_2_O)_2_]·2H_2_O}, [Cd_3_(L)_2_(bib)_2_(H_2_O)_2_] and [Cd_3_(L)_2_(idy)_2_], where bic = 3,6-bis(imidazol-1-yl)-9*H*-carbazole, bbi = 1,1′-(1,4-butanediyl)bis(imidazole), bib = 1,4-bis(1-imidazoly)benzene and idy = 2-imidazol-1-ylpyridine [Cd(opda)(mbib)(H_2_O)], [Cd(opda)(pbib)(H_2_O)], [Cd(ppda)(mbib)], and [Zn_2_(mpda)_2_(mbib)_2_]·2H_2_O, (H_2_opda = 1,2-phenylenediacetic acid, H_2_mpda = 1,3-phenylenediacetic acid, H_2_ppda = 1,4-phenylenediacetic acid, mbib = 1,3-bis(imidazolyl)benzene, pbib = 1,4-bis(imidazolyl)benzene) [Cd(BPDC)(BP4VA)·2DMF]*_n_* (9,10-bis((*E*)-2-(pyridin-4-yl)vinyl)anthracene (BP4VA), and biphenyl-4,4′-dicarboxylic acid (H_2_BPDC) [104,105,106]. All of these functionalized MOF-based probes showed excellent selectivity and fast response time against antibiotics. Moreover, inconspicuous effect caused by various substances (such as metal ions, acid, base, and some antibiotics other than fluoroquinolone) appeared in the fluorescence detection results when these MOFs were applied to fluoroquinolone antibiotic sensing; moreover, the MOFs sample after a one time detection experiment could be reused without a distinct loss of function. Gai et al. demonstrated that Zn(II)-based MOFs {[Zn_2_(bcob)(OH)(H_2_O)]·DMA}n (ROD-Zn1) and {[Zn(Hbcob)]·(solvent)}_n_ (ROD-Zn2) (H_3_bcob = 1,3-bis((4′-carboxylbenzyl)oxy)benzoic acid) with rod second building units (SBUs) were also expected to be applied for detecting and removing antibiotics existing in water [107]. In addition, fluorescent arrays formed by MOF functionalization can also be used as probes for the rapid and selective detection of antibiotics. For example, by encapsulating the gust dye 4-(4-diethylaminostyryl)-1-methylpyridinium (DEASM) into the frameworks of MOFs, a series of dynamic breathing LMOFs dual-emission fluorescent arrays were obtained by Xing et al. for antibiotics detection in water [108]. The resulting dual-emission fluorescent arrays show biphasic behavior with linear color tuning from green to red and controlled sensitivity to the ratiometric luminescence response to pH, supported by dimensionally relevant energy transfer. Moreover, luminescence intensity of a dual-emission fluorescent array has no distinct change with good photostability after two weeks of storage in air and deionized water. The results of a Boolean logic-gate strategy illustrate dual-emission fluorescent composites could be a well-defined logic device for monitoring and analyzing NZF levels with a simple and facile method. Xie et al. fabricated a Eu^3+^ and Tb^3+^ co-doped LMOF based ratiometric array for identification and determination of antibiotics [109]. The reaction between MOFs and different antibiotics made an impact on the ratio of fluorescence intensity at 545 nm and 616 nm (F_545_/F_616_), after these various responses were differentiated by principal component analysis (PCA), eight kinds of 25 antibiotics were effectively distinguished with the existence of interfering substances. Both high accuracy (98%) for the recognition of 48 unknown sample existed in the water and the outstanding quantitative ability for the mixture of antibiotics were demonstrated in this test. Finally, the sensor array was proved to be practical for actual sample analysis. This strategy not only provides an effective method for the comprehensive identification and detection of antibiotics, but also provides a new opportunity for the development of a sensor array based on ratio signal.

**Figure 15:**
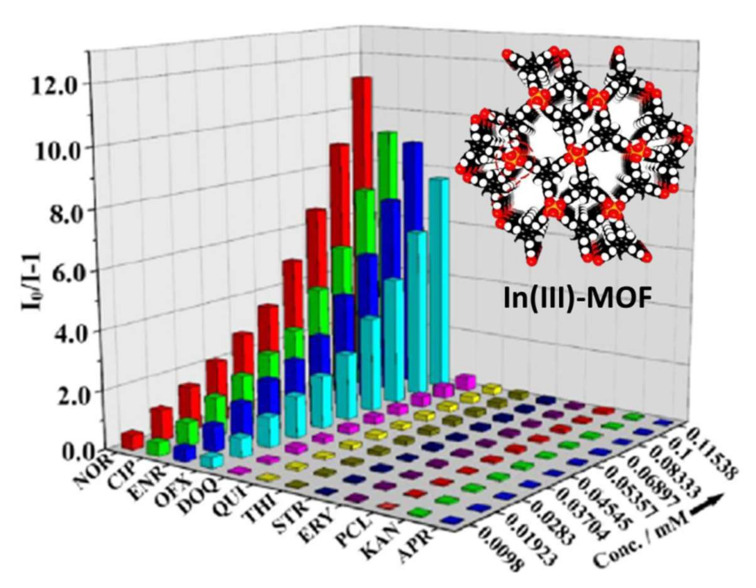
Quenching efficiencies of the tested antibiotics at different concentrations on the fluorescence of In(III)-MOF at room temperature; inset: the structure of In(III)-MOF. Adapted from [114].

Apart from nitrofuran antibiotics, LMOFs have also been used in the detection of tetracycline (TC), sulfonamide, fluoroquinolones, and nitroimidazole antibiotics [110]. In 2018, Zhou et al. explored the first example water-stable LMOF-based probe of Zr-based MOF (PCN-128Y) based on tetraphenylethylene (TPE)-based ligand (H_4_ETTC) for detecting TC in water, with an experimental detection limit of 30 nm [111]. Theoretical/experimental studies prove that the luminescence quenching can be attributed to a combined function of the strong absorption of TC at the excitation wavelength and the PIET process from the ligand of PCN-128Y to TC. In addition, other MOF-based probes have also been proposed for TC detection, such as nanosensor and hybrid heterostructures. Specifically, the energized moieties capable of forming host-guest interactions with TC have been widely used to integrate with MOFs. On the basis of the results noted above, Li et al. constructed a nano-MOF (Al-MOF@Mo/Zn-MOF) containing amino moiety [112]. The hydrogen-bonding and *π*–*π* interactions among Al-MOF@Mo/Zn-MOF and TCs can be formed due to the strong chelating capable of the metal nodes of Al, Zn, and Mo to the analytes. The luminescent intensities at 425 nm decreased gradually with the increase of TC content. The LOD of Al-MOF@Mo/Zn-MOF for DOX, TET, OTC, and CTC achieved 0.56, 0.53, 0.58, and 0.86 nm, respectively. These values are almost the lowest when compared to other reported MOF-based detectors. The quenching mechanism of Al-MOF@Mo/Zn-MOF participated in a PET process through H-bonding interaction and the competitive absorption of excitation energy. Zhu et al. proposed a LMOF, namely [Zn_3_(*μ*_3_-OH)(HL)L(H_2_O)_3_]·H_2_O (H_3_L = 5-(4-carboxy-phenoxymethyl)-isophthalic acid), with the character of highly ordered structure [113]. The experimental data showed that this MOF possesses great stability in simulated waste water and expectable sensitivity and rapid response to a series of sulfonamide antibiotics by fluorescence quenching as a biosensor. Using H_3_CTTA as organic linker (H_3_CTTA = 5′–(4-carboxyphenyl)-2′,4′,6′-trimethyl-[1,1′:3′,1′′-terphenyl]-4,4′′-dicarboxylic acid), another study fabricated an In(III)-MOF with rod-shaped SBU, which links the CTTA^3−^ linker into a 3D framework [114]. In(III)-MOF displayed an apparent emission at 380 nm when excited at 280 nm. The detected results showed that In(III)-MOF can selectively sensor fluoroquinolones (norfloxacin, ciprofloxacin, and enrofloxacin) with LOD of 56.7, 72.9, and 79.0 ppb, respectively, as shown in Figure 15. The UV–Vis spectra manifested that the absorption peak of these three antibiotics at 280 nm were high-degree overlapped the excitation band of In(III)-MOF. Therefore, the principle of the sensing process is likely to be involved in a competitive absorption and FRET among the MOF framework and the antibiotic. LMOF based sensors exhibited outstanding performance in antibiotic detection. However, because of the poor structure stability in water and the difficulty in pore modification, the device application of such materials is limited to some extent.

#### 3.3.2. Pesticides

Excessive and inappropriate use of pesticides in agricultural production results in high pesticide residue levels, which not only directly pollute water and the environment, but also seriously endangers human health [115]. Due to the high toxicity of pesticides, accurate and sensitive detection of pesticide residues has become imperative to protect the environment and food resources. The detection of pesticides has long been achieved using conventional methods such as gas chromatography (GC), high-performance liquid chromatography (HPLC), capillary electrophoresis, potentiometry, and flow injection spectrophotometry. However, use of these methods has often been limited by a number of disadvantages, including high analytical costs, time-consuming procedures (in sample preparation and pretreatment), and sophisticated instrumentation. In light of the limitations associated with the conventional methods, there has been a growing demand for quick and reliable methods for detection of various pesticides in environmental samples. This demand has been partially met using chromogenic and luminescent chemosensors.

Since one study in 2014 demonstrated the original case of an LMOF-based probe for pesticide detection with fast response and high selectivity, various MOF-based fluorescent probes have been used to detect different pesticides, in particular organophosphates (OPs) and non-organophosphates (non-OPs) [116]. The pesticides detection ability and detection mechanisms of various LMOF-based sensors are summarized in Entry 2 of Table 3.

##### Organophosphates (OPs)

Organophosphates are widely used in agriculture as a very important class of pesticides, generally including parathion, methyl parathion, and fenitrothion. The rapid and selective detection of OPs is significant due to their relatively high performance and moderate environmental persistence. The -NO groups in OPs can act as Lewis acid-base sites, interacting with the electron-rich centers of LMOFs, leading to luminescence detection. In 2014, Kumar et al. discussed the effect of a range of nitro-OPs on MOF-5 and found that all of the nitro-OPs similarly quenched the luminescence of MOF-5 [117]. In contrast, no quenching behavior was evident for other non-nitro organic compounds (e.g., malathion, dichlorvos, and monocrotophos). It was observed that the luminescence quenching of MOF-5 is related to the presence of nitro-organic groups in this solution. Subsequently, ref. [118] proposed a functionalised MOF to detect nitro-OPs of p-nitrophenols in water with a simultaneous detection limit of 0.36 μg mL^−1^. The luminescence spectra of the probe barely changed although the temperature varied from 15 °C to 30 °C. The possible mechanism is the competitive uptake among linkers and metabolites. Ma et al. obtained a Zr-based MOF acetylcholinesterase (AChE) biosensor with large surface area and high-dispersion Pt nanoparticles for the detection of Ops [119]. Due to the combined influence of electron conduction channels and the increase in adsorption dots as well as the ultra-high surface dimension to enhance AChE immobilization, the proposed biosensors demonstrated high sensitivity to malathion; the presented biosensors achieved the fairly low detected limit of 1 × 10^−^^14^ M to 1 × 10^−^^9^ M and 4.9 × 10^−^^15^ M. Fluorescent probes based on enzyme-mimicking activities are burgeoning as prospective candidates for the colorimetric detection of OPs.

**Figure 16:**
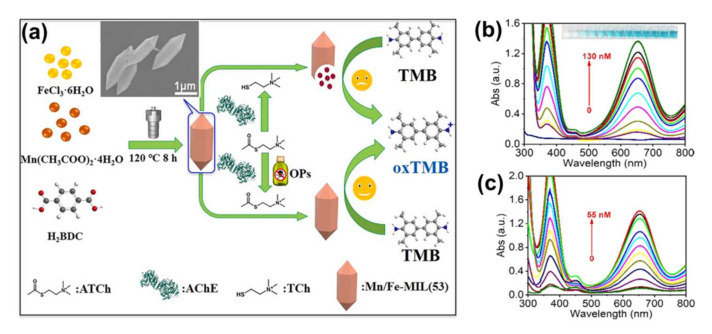
(**a**) Representation of the sensing process of Mn/Fe-MIL(53) MOF nanoenzyme for OPs detection. UV–vis spectrum of nanoenzyme-TMB catalyzed system corresponding to different dosages of methyl parathion (**b**) and clorpyrifos (**c**). Adapted from [121].

By integrating the enzymolysis product from AChE and choline oxidase (CHO) on AuNCs@ZIF-8, a dual function probe with a fluorescence lifetime and quantum yield of 6.83 μs and 4.63% was obtained by Cai et al. [120]. The authors used colorimetric strips constructed from the fluorescent material to visualise semi-quantitative detection. In addition, they developed a smartphone app to make the visualisation more accurate and enable real-time regulation of pesticide contamination. Similarly, Luo et al. introduced Mn ions into Fe-based MOF (Fe-MIL(53)) via a one-pot hydrothermal reaction strategy, resulting in a bimetallic Mn/Fe-MIL(53) MOF nanoenzyme that achieves selectivity and sensitivity for OPs detection, as shown in Figure 16a [121]. The proposed Mn/Fe-MIL(53) MOF nanozyme can be used for a quantitative analysis of methyl parathion and chlorpyrifos in the concentration range of 10-120 nM and 5-50 nM, respectively. The detection limits of 0.95 nM (3 S/N) for chlorpyrifos and 2.8 nM for methyl parathion were also realized, as shown in Figure 16b,c. Good recoveries were obtained when applied in actual sample assays. In addition, this work establishes a solid foundation for improving the catalytic performance of MOF nanozymes, which is of great significance for biosensing.

##### Nonorganic Phosphates (Non-OPs)

**Figure 17:**
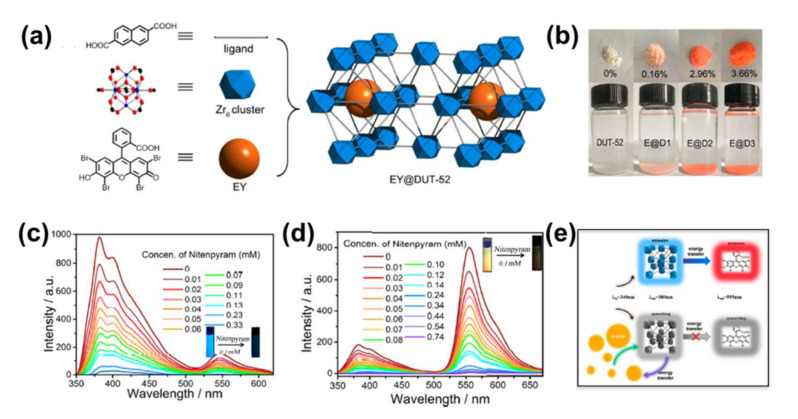
(**a**) Structure diagram of the as-prepared EY@DUT-52 sample. (**b**) Optical pictures of DUT-52 and three EY@DUT-52 samples under sunlight (the solvent in the vial is ethanol). (**c**) Fluorescent emission spectra of E@D1/ethanol suspensions with different concentrations of nitenpyram. (**d**) Fluorescent emission spectra of E@D3/ethanol suspensions with different concentrations of nitenpyram. Adapted from [127].

Non-OP pesticides are general insecticides, including glyphosate, paraquat, endiquat, and so on. Wiwasuku et al. constructed a microcrystalline Cd(II)-MOF enhanced with open amino and azo agents, called [Cd(NH_2_-bdc)(azp)]·DMF (NH_2_-H_2_bdc = 2-amino-1,4-benzenedicarboxylic acid, azp = 4,4′-azopyridine), as a fluorescent sensor in the off state using a PET process [122]. As the first MOF-based probe for detecting non-OP pesticides, [Cd(NH_2_-bdc)(azp)]·DMF shows highly selective and sensitive visual turn-on fluorescence detection of glyphosate and Cr^3+^ with LOD of 25 nM and 0.6 μM, respectively. The amino group in [Cd(NH_2_-bdc)(azp)]·DMF plays a key role in the selective binding of Cr^3+^, facilitating the sensitivity and selectivity of the detection. DFT indicated that the mechanism of fluorescence enhancement was related to the inhibition of the PET process stimulated by the framework of [Cd(NH_2_-bdc)(azp)]·DMF dissociation in the presence of glyphosate and Cr^3+^. Recently, Li et al. reported a case of a water-stable 3D MOF, [Tb(L)_2_NO_3_]_n_ (HL = 3.5-bis(triazol-1-yl)benzoic acid), that can distinguish various pesticides under a 2D decoding map with ratio fluorescence [123]. [Tb(L)_2_NO_3_]_n_ is an ideal fluorescent probe for the detection of glyphosate with high selectivity and sensitivity. The detection limit for glyphosate was 0.0144 μM. In addition to glyphosate, LMOF-based sensors can also selectively detect paraquat in aqueous systems. For example, Chen et al. constructed a bifunctional LMOF-based sensor [Zn_2_(cptpy)(btc)(H_2_O)]_n_ for paraquat detection in water by hydrothermal reaction of Zn^2+^ ions with a mixture of two ligands, Hcptpy and H_3_btc (Hcptpy = 4-(4-carboxyphenyl)-2,2′:4′,4′′-terpyridine; H_3_ btc = 1,3,5-benzenetricarboxylic acid) [124]. This bifunctional LMOF-based sensor could be stable in air for more than 3 months and in aqueous solutions (pH 3–11) for at least 1 week. Parmar et al. evaluated Zn(II)/Cd(II) based luminescent MOFs/CPs with mixed ligands to address problems associated with water pollution [125]. The advantages of Zn(II) and Cd(II) metal ions, the hybrid linker system, and the subtle host-guest interactions between the framework and analyte allow this fluorescent system to detect organic contaminants. Zhao et al.has developed a pyrene-based MOF probe with anionic ligands for the analysis of paraquat in aqueous and organic phases. The electrostatic attraction between the anionic ligands in the framework of the pyrene-based MOF probe improves the sensing performance [126]. In addition, the system achieved the identification of paraquat in a range of electron deficient agrochemicals and reached the detection limit at the nM level. Wei et al. obtained an EY@Zr-MOF for detecting nitenpyram in which the fluorescent molecule of eosin Y (EY) was implanted into Zr-MOF. EY@Zr-MOF manifested the dual emission signals emitted by the Zr-MOF and EY molecules at 430 and 560 nm, respectively, as shown in Figure 17a [127]. The colors of EY@Zr-MOF could be gradually transformed from bright brown to orange when increasing the loading molar ratio of EY, leading to E@D1 (0.023 mol%), E@D2 (0.18 mol%), and E@D3 (0.22 mol%), as shown in Figure 17b. After the mixture of 20 pesticides, both E@D1 and E@D3 exhibited selective detection of nitenpyram by the changes in luminescence ratios, as shown in Figure 17c,d.

#### 3.3.3. Nitro-Explosives

Nitro explosives, such as 2,4,6-trinitrophenol (TNP), 2,4,6-trinitrotoluene (TNT), cyclotrimethylene trinitramine (RDX), 2,4-dinitrotoluene (2,4-DNT), and so on, are widely used in military production and industrial blasting. These compounds are not only explosive, but also toxic, hard to degrade, carcinogenic, and can cause vomiting, convulsions, neurological disorders, and even death after introduction into the human body [128,129]. Therefore, the development of rapid trace detection technology for nitro explosives is of great significance to prevent terrorist attacks, maintain public safety, and safeguard human health. However, the low vapor pressure and chemical reactivity make nitro explosives difficult to be detected. Current detection methods typically involve canines or sophisticated instruments. Both techniques are expensive and may not always be easily accessible. Fluorescence quenching employing conjugated polymers is a simple and promising alternative procedure that is based on the donor-acceptor electron-transfer mechanism. 

**Figure 18:**
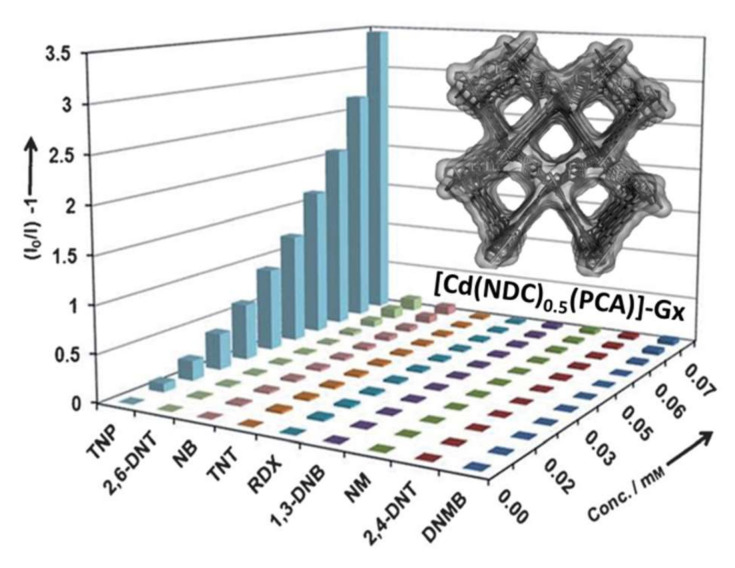
Quenching efficiencies of tested nitro-explosives at different concentrations on the fluorescence of [Cd(NDC)_0.5_(PCA)]-Gx at room temperature; inset: the structure of [Cd(NDC)_0.5_(PCA)]-Gx. Adapted from [133].

To date, various LMOFs have already been proposed to detect explosives through PET and FRET mechanisms, where the electrostatic interactions and electron transfer perform a significant role in the explosives sensing process [130]. The nitro-explosive detection ability and detection mechanisms of various LMOF-based sensors are summarized in Entry 3 of Table 3. In 2009, the first example of using LMOF to detect explosives was reported by Lan et al., namely [Zn_2_(bpdc)_2_(bpee)], providing a new application of microporous MOFs [131]. Subsequently, in order to diversify aromatic explosives with electron-withdrawing groups or electron-donating groups in the vapor, they constructed another microporous LMOF of [Zn_2_(oba)_2_(bpy)]·3DMA [132]. The detection mechanism is also given, which shows that only explosives with nitro electron-withdrawing groups can significantly decrease the luminescence intensity of Zn-MOF. On photo-excitation, the energy in the conduction band (CB) of Zn-MOF and the LUMO band of the explosive can produce electron transfer in the explosives sensing process. If explosives with electron withdrawing groups are present, a quenching effect occurs, which is based on the transfer of electrons from the CB of Zn-MOF to the LUMO of nitroaromatics. Importantly, this work proves that the detection capability is related to suitable porosity, strong overlap, and the driving force for electron transfer between the LMOF and analyte molecules. Since then, more and more scientists are focusing on detecting explosives using LMOF-based sensors. One study verified the selective detection of TNP by the MOF-based sensor for the first time; [Cd(NDC)_0.5_(PCA)]-Gx (G = guest molecule, NDC = 2,6-naphthalenedicarboxylic acid, PCA = 4-pyridinedicarboxylic acid) showed a strong emission at 384 nm when it was excited at 340 nm [133]. Luminous intensity of this Cd-MOF can be quenched by nearly 78% when dispersed in TNP solution, as shown in Figure 18. Importantly, this work allows for the detection of explosives in aqueous solutions, showing great application prospect in the future.

In addition to the sensing principle of energy transfer in LMOFs, the AIE mechanism was also used to sense explosives [134]. Guo et al. produced three M-TABD-MOFs (M = Mg^2+^, Ni^2+^, and Co^2+^) using TABD-COOH (4,4-(Z,Z)-1,4-diphenylbutane-1,3-diene-1,4-diyl) dibenzoyl as AIE linkers [135]. Usually, the coordination between metal and carboxylate agent would be weakened by the heterocyclic explosive, which contains C=N and/or N=N band, and as a result, partial linkers will be replaced. Thus, MOFs sensors with AIE connectors for the detection of explosives can be designed where the AIE molecules can be released and reassembled to form emissive aggregates. Because of the emulative replacement of the ligand, it is still hard to maintain the integrity of the framework TABD-MOF after the mixture of NTO (5-nitro-2,4-dihydro-3H-1,2,4-triazol-3-one). Therefore, to release free TABD-COOH molecules into solution, trace amounts of TABD-MOFs can be deposited on the paper strips. The dissociated molecules TABD-COOH can be aggregated again after evaporation of the THF solvent to enable sustainable detection.

#### 3.3.4. Hazardous Solvents

Hazardous solvents, highly toxic substances that can cause cancer, deformities, and blood poisoning, generally include toxic organic solvents and amine-based organic molecules [136,137]. Up to now, several methods such as high performance liquid chromatography, nuclear magnetic resonance spectroscopy, mass spectrometry, and infrared and ultraviolet (UV) spectroscopy have been available for probing hazardous solvents. Recently, MOFs as luminescent sensors to recognize acetylacetone have also been explored. The hazardous solvents detection ability and detection mechanisms of various LMOF-based sensors are summarized in Entry 4 of Table 3. Early in 2011, Lu et al. produced a solvatochromic MOF {[(WS_4_Cu_4_)I_2_(dptz)_3_]·3DMF}_n_ (dptz) for sensing solvents [138]. They found that the absorption bands of {[(WS_4_Cu_4_)I_2_(dptz)_3_]·3DMF}_n_ were shifted to blue in UV–Vis spectra when the polarity of the solvent increases.

**Figure 19:**
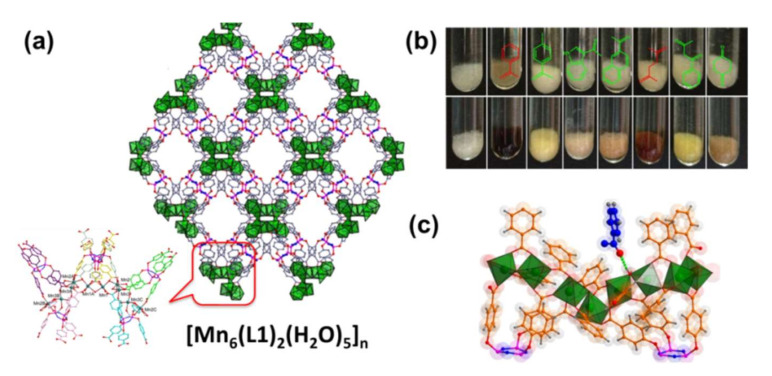
(**a**) Structure of [Mn_6_(L_1_)_2_(H_2_O)_5_]_n_. (**b**) Photographs of different solvent-loaded [Mn_6_(L_1_)_2_(H_2_O)_5_]_n_. (**c**) Perspective view of the hydrogen-bonding interaction between the acetophenone molecule (blue color) and host framework. Adapted from [139].

Subsequently, a growing number of LMOF-based probes have been exploited for the identification of poisonous organic solvents. For example, Li et al. reported a 3D porous MOF of [Mn_6_(L_1_)_2_(H_2_O)_5_]_n_, as shown in Figure 19a [139]. Solvent recognition experiments showed that the color variations of this MOF could be distinctly observed with the naked eye when treated with ketone molecules, as shown in Figure 19b. The possible induction mechanism can be attributed to the hydrogen bonding interaction between guest ketone molecules and μ_2_-bridged oxygen, which can lead to the deformation of the octahedral coordinated Mn^2+^ center, as shown in Figure 19c. Li et al. developed an ultra-stable tetrazolium Cd-based MOF {[Cd_2_(μ_7_-L)(μ_3_-OH)(H_2_O)_2_]·1.3H_2_O}_n_ containing a new bi-functional ligand H_3_L (H_3_L = 4′-(1H-tetrazole-5)-biphenyl-2,3-dicarboxylic acid) under the solvo-thermal conditions [140]. Fluorescent characterization shows that this ultra-stable MOF allows highly sensitive real-time discrimination of acetylacetone with good reusability (high quenching efficiency *K*_sv_ for acetylacetone: 2.5 × 10^5^ M^−1^) and very low detection limit values (for acetylacetone: 35 μM). Recently, Ghosh et al. obtained a Zr^IV^-based UiO-66 LMOF with sulfonamide functionality for the detection of acetylacetone [141]. The thermally activated MOF showed highly selective and sensitive fluorescent turn-on properties for superoxide (O_2_^•−^) in MeOH and acetylacetone in DMF medium. The detection limit (98 nM) of O_2_^•−^ using this probe is much lower than that of known MOF-based chemical sensors for O_2_^•−^. The MOF is unique in that it is able to detect acetylacetone up to the limit of 1.23 ppm, which is similar to that of the previously published MOF chemo-sensors for acetylacetone.

**Figure 20:**
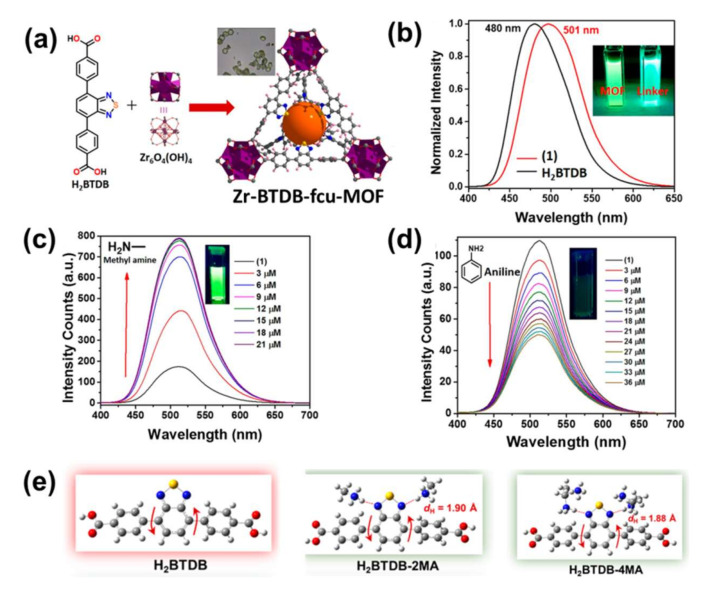
(**a**) Schematic representation of the synthesis of Zr-BTDB-fcu-MOF (1). (**b**) PL spectra for (1) (red) and linker (black). (**c**) Fluorescence intensity of (1) aqueous suspension upon addition of 3 μM of of MA (**c**), aniline (**d**) (λmax = 515 nm). (**e**) Molecular structures of H_2_BTDB, H_2_BTDB with two and four MA molecules. Red arrows represent the dihedral angles between benzene and thiadiazole-based units, and red dashed lines indicate the hydrogen-bond lengths (dH) formed by H of MA and N of thiadiazole Adapted from [146].

In addition to ketones, ammonia solvents can also be detected by LMOF-based probes [142,143,144]. For example, one study designed a porous MOF of Mg-NDI using chromophore naphthalenediimide (NDI) for detecting solid-state organic amine (like aniline, ethylenediamine, triethylamine, and hydrazine) [145]. The rapid color change from yellow to dark black of Mg-NDI also can be seen by the naked eye. A further study achieved the detection of aliphatic amines in aqueous solution by Zr-BTDB fcu-MOF (H_2_BTDB = 4,4′-(2,1,3-benzothiadiazole-4,7-diyl) bis[benzoic acid]) based on the underlying fcu topology, as shown in Figure 20a,b [146]. Due to the strong hydrogen bonding between protonated methylamine and N atoms of thiadiazole, the dihedral angle (thiadiazole unit and benzene) of the linker (H_2_BTDB) becomes smaller from 22 to 12 when H_2_BTDB is attached to two or four methylamine, as shown in Figure 20c,d. Therefore, this hydrogen bond can inhibit the rotation of thiadiazole core in linker H_2_BTDB and reduces the pathway for non-radiative recombination. Importantly, the hydrogen bond interaction between ligands and amines results in an ultra-low detection limit of 66 nm, as shown in Figure 20e.

**Table 3:** The hazardous organic pollutant detection ability and detection mechanisms of various LMOF-based sensors.

Entries	Category	MOF	Sensing Target	Detection Limit	Detection Mechanism	Ref
Entry 1	Antibiotics	Zr(IV)-MOF	NZF TNP	58 ppb 90 ppb	PET FRET	[101]
		[Cd_2_Na(L)(BDC)_2.5_]·9H_2_O [Cd_2_(L)(2,6-NDC)_2_]·DMF·5H_2_O [Cd_2_(L)(BPDC)_2_]·DMF·9H_2_O	NZF	162 ppb 75 ppb 60 ppb	PET FRET	[102]
		Cd-based LMOF	NFZ NFT	0.20µM 0.26 µM	PET	[103]
		[Cd(opda)(mbib)(H_2_O)] [Cd(opda)(pbib)(H_2_O)] [Cd(ppda)(mbib)] [Zn_2_(mpda)_2_(mbib)_2_]·2H_2_O	CEF TEC	0.278 μM for CEF; 0.384 μM for TEC 0.379 μM for CEF; 0.0.189 μM for TEC 0.52 μM for CEF; 0.421 μM for TEC 0.171 μM for CEF; 0.137 μM for TEC	PET FRET	[104]
		{[Cd_3_(L)_2_(bbi)_2_(H_2_O)_2_]·2H_2_O}	NZF	1.83 ppM	FRET	[105]
		{[Zn_2_(bcob)(OH)(H_2_O)]· DMA}n (ROD-Zn1) {[Zn(Hbcob)]·(solvent)}n (ROD-Zn2)	TC	0.11 μmol 0.12 μmol	PET FRET	[107]
		Eu^3+^ and Tb^3+^ co-doped LMOFs	Minocycline Norfloxacin	1.23 μM 0.06 μM	FRET PET	[109]
		Zr-based MOF (PCN-128Y)	TC	30 nM	FRET PET	[111]
		Al-MOF@Mo/Zn-MOF	DOX TET OTC CTC	0.56 nM 0.53 nM 0.58 nM 0.86 nM	FRET PET	[112]
		[Zn_3_(*μ*_3_-OH)(HL)L(H_2_O)_3_]· H_2_O	Sulfonamide	-	PET	[113]
		In(III)-MOFs	Norfloxacin Enrofloxacin Ciprofloxacin	56.7 ppb 79.0 ppb 72.9 ppb	FRET PET	[114]
Entry 2	Pesticides	MOF-5	Nitro-OPs	5 ppb	-	[117]
		Eu^3+^@1	p-nitrophenol 3-methyl-4-nitrophenol	0.36 μg mL^−1^ 0.41 μg mL^−1^	Competitive absorption	[118]
		Zr-based MOF	Malathion	4.9 × 10^−^^15^ M	-	[119]
		AuNCs@ZIF-8	OPs	0.3 μg/L	-	[120]
		Mn/Fe-MIL(53)	Methyl parathion Chlorpyrifos	2.8 nM 0.95 nM	-	[121]
		[Cd(NH_2_-bdc)(azp)]·DMF	Glyphosate	25 nM	Structural collapse of LMOFs	[122]
		[Tb(L)_2_NO_3_]_n_	Glyphosate	0.0144 μM	PET	[123]
		[Zn_2_(cptpy)(btc)(H_2_O)]_n_	Paraquat	9.73 × 10^−^^6^ mol/L	FRET	[124]
		NU-901 NU-901-sbdc	Paraquat	2.0 nM 3.3 nM	PET	[126]
		E@D1 E@D3	Nitenpyram	0.94 μM 1.18 μM	PET	[127]
Entry 3	Nitro- Explosives	[Zn_2_(oba)_2_(bpy)]·3DMA	Nitrobenzene	-	PET FRET	[132]
		[Cd(NDC)_0.5_(PCA)]-Gx	TNP	3.5 × 10^4^ M^−1^	PET FRET	[133]
		M-TABD-MOFs	NTO	4 × 10^−^^8^ mol/L	Structural collapse of LMOFs	[135]
Entry 4	Hazardous solvents	[Mn_6_(L_1_)_2_(H_2_O)_5_]_n_	Acetophenone	-	QD	[139]
		{[Cd_2_(μ_7_-L)(μ_3_-OH)(H_2_O)_2_]·1.3H_2_O}_n_	Aacetylacetone	35 μM	QD	[140]
		Zr^IV^-based UiO-66	Aacetylacetone	1.23 ppm	Object exchange PET	[141]
		Mg-NDI	Organic amine	-	PET	[145]
		Zr-BTDB fcu-MOF	Methylamine	66 nM	-	[146]

## 4. Conclusions and Outlook

Excessive emissions of toxic and hazardous substances have become a pressing global issue, as these highly soluble and mobile substances can easily spread into the atmosphere, land, and water, causing a serious threat to ecosystems. All current technologies for the sensing of toxic and hazardous substances have their own advantages and limitations due to their specific sensing applications. Recent years have seen that MOFs-based fluorescent probes have shown outstanding advantages in the fields of hazardous substance identification and detection. This study presents a summary of some of the latest developments in LMOF-based chemical sensors for detecting harmful gases, heavy metal ions, radioactive ions, and a range of harmful organic pollutants, with an emphasis on the influence of composition or structure on the sensing performance of MOF-based probes. A variety of luminescence sensing mechanisms and structure-performance relationships are also outlined. Despite significant advances and outstanding achievements of LMOF based sensors that have been achieved, there are still many challenges that need to be addressed.

First, adding the structural/luminescence stability of LMOFs in environmental media is a challenging mission, as hazardous substances are mostly discharged into relatively sophisticated environmental media. Water and air stability are prerequisites for MOF-based sensors to detect hazardous substances, which will ensure structural integrity and recoverability in sewage systems or complex environments with uncompromised performance. So far, very few families of MOFs such as the ZIFs, Al-based MOFs, MIL MOFs, and Zr-based MOFs, made up of high-valent metallic centers, imidazolate, terephthalate, and additional hydrophobic linkers based LMOFs, have demonstrated exceptional stability under wet conditions. Significant attempts within the MOF community to improve MOF stability include the construction of MOF composites with hydrophobic materials having special stability even under extreme acidic and alkaline environments. Second, a further developing characterization technique to verify and validate each of mechanism outlined above is beneficial for the potential of suggesting and developing new mechanisms. The exact synthesis methods, the in situ monitoring techniques, and the theoretical computations should be synergized to learn the structure–function relationships between LMOFs and the corresponding sensing performances. Third, more wearable and hand-held MOF sensors should be developed to enable fast and easy detection of diverse analytes as future MOF devices may play a key role in the revolution in healthcare, smart farming, environmental monitoring, defense, and artificial intelligence.

Briefly, in spite of the various challenges, MOFs still remain one of the most attractive classes of sensor materials, with great potential for applications in the detection of hazardous substances. With sensible design and optimization, MOF-based materials will continue to progress, offering unbelievable opportunities not only in the sensing field, but in numerous other fields as well.

## References

[1] Safaei M., Foroughi M.M., Ebrahimpoor N., Jahani S., Omidi A., Khatami M. (2019). A review on metal-organic frameworks: Synthesis and applications. Trends Anal. Chem..

[2] Rasheed T., Li C., Bilal M., Yu C., Iqbal H.M.N. (2018). Potentially toxic elements and environmentally-related pollutants recognition using colorimetric and ratiometric fluorescent probes. Sci. Total Environ..

[3] Kowalska J.B., Mazurek R., Gąsiorek M., Zaleski T. (2018). Pollution indices as useful tools for the comprehensive evaluation of the degree of soil contamination—A review. Environ. Geochem. Health.

[4] Eisenman T.S., Churkina G., Jariwala S.P., Kumar P., Lovasi G.S., Pataki D.E., Weinberger K.R., Whitlow T.H. (2019). Urban trees, air quality, and asthma: An interdisciplinary review. Landsc. Urban Plan..

[5] Mako T.L., Racicot J.M., Levine M. (2018). Supramolecular luminescent sensors. Chem. Rev..

[6] Nguyen M.P., Kelly S.P., Wydallis J.B., Henry C.S. (2020). Read-by-eye quantification of aluminum (III) IN distance-based microfluidic paper-based analytical devices. Anal. Chim. Acta.

[7] Cai X., Xie Z., Li D., Kassymova M., Zang S.-Q., Jiang H.-L. (2020). Nano-sized metal-organic frameworks: Synthesis and applications. Coord. Chem. Rev..

[8] Yang J., Ni W., Ruan B., Tsai L.-C., Ma N., Shi D., Jiang T., Tsai F.C. (2021). Review-design and synthesis of fluorescence sensing metal-organic frameworks. ECS J. Solid State Sci. Technol..

[9] Cui Y., Zhang J., He H., Qian G. (2018). Photonic functional metal–organic frameworks. Chem. Soc. Rev..

[10] McDonagh C., Burke C.S., MacCraith B.D. (2008). Optical chemical sensors. Chem. Rev..

[11] Zhang D.S., Gao Q., Chang Z., Liu X.T., Zhao B., Xuan Z.H., Hu T.L., Zhang Y.H., Zhu J., Bu X.H. (2018). Rational construction of highly tunable donor–acceptor materials based on a crystalline host–guest platform. Adv. Mater..

[12] Zhao D., Liu X.-H., Guo J.-H., Xu H.-J., Zhao Y., Lu Y., Sun W.-Y. (2018). Porous metal−organic frameworks with chelating multiamine sites for selective adsorption and chemical conversion of carbon dioxide. Inorg. Chem..

[13] Zhang Y., Yuan S., Day G., Wang X., Yang X., Zhou H.-C. (2018). Luminescent sensors based on metal-organic frameworks. Coord. Chem. Rev..

[14] Dong J., Zhao D., Lu Y., Sun W.-Y. (2019). Photoluminescent metal–organic frameworks and their application for sensing biomolecules. J. Mater. Chem. A.

[15] Bai X., Yuan D., Xu Y., Li Y., Liu W., Wang S., Fu G., Hu Y. (2020). Realizing efficient natural sunlight–driven photothermal selective catalytic reduction of nitrogen oxides by AlN_x_ assisted W doped Fe_2_O_3_ nanosheets. Sol. Energy Mat. Sol. C..

[16] Bünzli J.C.G. (2006). Benefiting from the unique properties of lanthanide ions. Acc. Chem. Res..

[17] Dolgopolova E.A., Rice A.M., Martin C.R., Shustova N.B. (2018). Photochemistry and photophysics of MOFs: Steps towards MOF–based sensing enhancements. Chem. Soc. Rev..

[18] Carter K.P., Young A.M., Palmer A.E. (2014). Fluorescent sensors for measuring metal ions in living systems. Chem. Rev..

[19] Zhang H., Lin C., Sheng T., Hu S., Zhuo C., Fu R., Wen Y., Li H., Su S., Wu X. (2016). A luminescent metal–organic framework thermometer with intrinsic dual emission from organic lumophores. Chem.-Eur. J..

[20] Guo M.Y., Li P., Yang S.L., Bu R., Piao X.-Q., Gao E.-Q. (2020). Distinct and selective amine-and anion-responsive behaviors of an electron-deficient and anion-exchangeable metal-organic framework. ACS Appl. Mater. Inter..

[21] Zhao Y., Zeng H., Zhu X.W., Lu W., Li D. (2021). Metal–organic frameworks as photoluminescent biosensing platforms: Mechanisms and applications. Chem. Soc. Rev..

[22] Kreno L.E., Leong K., Farha O.K., Allendorf M., Van Duyne R.P., Hupp J.T. (2012). Metal-organic framework materials as chemical sensors. Chem. Rev..

[23] Yang G.-L., Jiang X.-L., Xu H., Zhao B. (2021). Applications of MOFs as luminescent sensors for environmental pollutants. Small.

[24] Bongaarts J. (1992). Population growth and global warming. Popul. Dev. Rev..

[25] Pales J.C., Keeling C.D. (1965). The concentration of atmospheric carbon dioxide in Hawaii. J. Geophys. Res..

[26] Gheorghe A., Lugier O., Ye B., Tanase S. (2021). Metal–organic framework based systems for CO_2_ sensing. J. Mater. Chem. C.

[27] Qi X.-L., Lin R.-B., Chen Q., Lin J.-B., Zhang J.-P., Chen X.-M. (2011). A flexible metal azolate framework with drastic luminescence response toward solvent vapors and carbon dioxide. Chem. Sci..

[28] Tan Y., Chen J., Wu H., Yu J., Jia J., Xu W., Fu Y., He Q., Cao H., Cheng J. (2020). A highly fluorescent post-modified metal organic framework probe for selective, reversibleand rapid carbon dioxide detection. Dyes Pigments.

[29] Montoya L.A., Pluth M.D. (2012). Selective turn-on fluorescent probes for imaging hydrogen sulfide in living cells. Chem. Commun..

[30] Guo L., Wang M., Cao D. (2018). A novel Zr-MOF as fluorescence turn-on probe for real-time detecting H_2_S gas and fingerprint identification. Small.

[31] Vikrant K., Kailasa S.K., Tsang D.C.W., Lee S.S., Kumar P., Giri B.S., Singh R.S., Kim K.H. (2018). Biofiltration of hydrogen sulfide: Trends and challenges. J. Cleaner Prod..

[32] Vikrant K., Kumar V., Ok Y.S., Kim K.H., Deep A. (2018). Metal-organic framework (MOF)-based advanced sensing platforms for the detection of hydrogen sulfide. Trends Anal. Chem..

[33] Hao Y., Chen S., Zhou Y., Zhang Y., Xu M. (2019). Recent progress in metal–organic framework (MOF) based luminescent chemodosimeters. Nanomaterials.

[34] Liu G., Cadiau A., Liu Y., Adil K., Chernikova V., Carja L.-D., Belmabkhout Y., Karunakaran M., Shekhah O., Zhang C. (2018). Enabling fluorinated MOF-based membranes for simultaneous removal of H_2_S and CO_2_ from natural gas. Angew. Chem. Int. Edit..

[35] Ma Y., Zhang C., Yang P., Li X., Tong L., Huang F., Yue J., Tang B. (2018). A CuO-functionalized NMOF probe with a tunable excitation wavelength for selective detection and imaging of H_2_S in living cells. Nanoscale.

[36] Ghosh S., Biswas S. (2021). Ultrafast and nanomolar level detection of H_2_S in aqueous medium using a functionalized UiO-66 metal–organic framework based fluorescent chemosensor. Dalton Trans..

[37] Nagarkar S.S., Saha T., Desai A.V., Talukdar P., Ghosh S.K. (2014). Metal-organic framework based highly selective fluorescence turn-on probe for hydrogen sulphide. Sci. Rep..

[38] Nagarkar S.S., Desai A.V., Ghosh S.K. (2015). A nitro-functionalized metal–organic framework as a reaction-based fluorescence turn-on probe for rapid and selective H_2_S detection. Chem. Eur. J..

[39] Zhang X., Zhang J.M., Hu Q., Cui Y.J., Yang Y., Qian G.D. (2015). Postsynthetic modification of metal–organic framework for hydrogen sulfide detection. Appl. Surf. Sci..

[40] Buragohain A., Biswas S. (2016). Cerium-based azide- and nitro-functionalized UiO-66 frameworks as turn-on fluorescent probes for the sensing of hydrogen sulphide. CrystEngComm.

[41] Nandi S., Reinsch H., Banesh S., Stock N., Trivedi V., Biswas S. (2017). Rapid and highly sensitive detection of extracellular and intracellular H_2_S by an azide-functionalized Al (III)-based metal–organic framework. Dalton Trans..

[42] Dalapati R., Balaji S.N., Trivedi V., Khamari L., Biswas S. (2017). A dinitro-functionalized Zr (IV)-based metal-organic framework as colorimetric and fluorogenic probe for highly selective detection of hydrogen sulphide. Sens. Actuators B Chem..

[43] Das A., Banesh S., Trivedi V., Biswas S. (2018). Extraordinary sensitivity for H_2_S and Fe (III) sensing in aqueous medium by Al-MIL-53-N_3_ metal–organic framework: In vitro and in vivo applications of H_2_S sensing. Dalton Trans..

[44] Nandi S., Banesh S., Trivedi V., Biswas S. (2018). A dinitro-functionalized metal–organic framework featuring visual and fluorogenic sensing of H_2_S in living cells, human blood plasma and environmental samples. Analyst.

[45] Chi K.N., Guan Y., Zhang X., Yang T., Meng S., Hu R., Yang Y.H. (2021). Iodide/metal-organic frameworks (MOF)-mediated signal amplification strategy for the colorimetric detection of H_2_O_2_, Cr_2_O_7_^2−^ and H_2_S. Anal. Chim. Acta.

[46] Zhang X., Zhang Q., Yue D., Zhang J., Wang J.T., Li B., Yang Y., Cui Y.J., Qian G.D. (2018). Flexible metal–organic framework-based mixed-matrix membranes: A new platform for H_2_S Sensors. Small.

[47] Zhang X., Hu Q., Xia T., Zhang J., Yang Y., Cui Y., Chen B., Qian G. (2016). Turn-on and ratiometric luminescent sensing of hydrogen sulfide based on metal–organic frameworks. ACS Appl. Mater. Interfaces.

[48] Zhang X., Fang L., Jiang K., He H., Yang Y., Cui Y., Li B., Qian G. (2019). Nanoscale fluorescent metal–organic framework composites as a logic platform for potential diagnosis of asthma. Biosens. Bioelectron..

[49] Yang X.L., Cheng D., Guan R.F., Zhang W.H., Feng Y., Xie M.H. (2021). Selective dual detection of H_2_S and Cu^2+^ by a post-modified MOF sensor following a tandem process. J. Hazard. Mater..

[50] Tchalala M.R., Bhatt P.M., Chappanda K.N., Tavares S.R., Adil K., Belmabkhout Y., Shkurenko A., Cadiau A., Heymans N., De Weireld G. (2019). Fluorinated MOF platform for selective removal and sensing of SO_2_ from flue gas and air. Nat. Commun..

[51] Chernikova V., Yassine O., Shekhah O., Eddaoudi M., Salama K.N. (2018). Highly sensitive and selective SO_2_ MOF sensor: The integration of MFM-300 MOF as a sensitive layer on a capacitive interdigitated electrode. J. Mater. Chem. A.

[52] Wang M., Guo L., Cao D. (2018). Amino-functionalized luminescent metal–organic framework test paper for rapid and selective sensing of SO_2_ gas and its derivatives by luminescence turn-on effect. Anal. Chem..

[53] Wang L., Chen Y. (2020). A reaction-triggered luminescent Ce^4+^/Tb^3+^ MOF probe for the detection of SO_2_ and its derivatives. Chem. Commun..

[54] Small L.J., Henkelis S.E., Rademacher D.X., Schindelholz M.E., Krumhansl J.L., Vogel D.J., Nenoff T.M. (2020). Near-zero power MOF-based sensors for NO_2_ detection. Adv. Funct. Mater..

[55] Moscoso F.G., Almeida J., Sousaraei A., Lopes-Costa T., Silva A.M.G., Cabanillas-Gonzalez J., Cunha-Silva L., Pedrosa J.M. (2020). Luminescent MOF crystals embedded in PMMA/PDMS transparent films as effective NO_2_ gas sensors. Mol. Syst. Des. Eng..

[56] Jo Y.-M., Lim K., Yoon J.W., Jo Y.K., Moon Y.K., Jang H.W., Lee J.-H. (2021). Visible-light-activated type II heterojunction in Cu_3_ (hexahydroxytriphenylene)_2_/Fe_2_O_3_ hybrids for reversible NO_2_ sensing: Critical role of π–π* transition. ACS Cent. Sci..

[57] Li Z., Zhang Y., Zhang H., Jiang Y., Yi J.X. (2020). Superior NO_2_ sensing of MOF-derived indium-doped ZnO porous hollow cages. ACS Appl. Mater..

[58] Wu P., Wang J., He C., Zhang X., Wang Y., Liu T., Duan C. (2012). Luminescent metal-organic frameworks for selectively sensing nitric oxide in an aqueous solution and in living cells. Adv. Funct. Mater..

[59] Desai A.V., Samanta P., Manna B., Ghosh S.K. (2015). Aqueous phase nitric oxide detection by an amine-decorated metal–organic framework. Chem. Commun..

[60] Schulz M., Gehl A., Schlenkrich J., Schulze H.A., Zimmermann S., Schaate A. (2018). A calixarene-based metal–organic framework for highly selective NO_2_ detection. Angew. Chem. Int. Edit..

[61] Gamonal A., Sun C., Mariano A.L., Fernandez-Bartolome E., Guerrero-SanVicente E., Vlaisavljevich B., Castells-Gil J., Marti-Gastaldo C., Poloni R., Wannemacher R. (2020). Divergent adsorption-dependent luminescence of amino-functionalized lanthanide metal–organic frameworks for highly sensitive NO_2_ Sensors. J. Phys. Chem. Lett..

[62] WaqasKhan M., MunirSadiq M., Gopalsamy K., Xu K., Jannat A., Zhang B.Y., Mohiuddin M., Haris M., Ou R., Afrin S. (2022). Hetero-metallic metal-organic frameworks for room-temperature NO sensing. J. Colloid Interface Sci..

[63] Zhu S.Y., Yan B. (2018). Highly sensitive luminescent probe of aniline and trace water in organic solvents based on covalently modified lanthanide metal-organic frameworks. Ind. Eng. Chem. Res..

[64] Liu W., Chen N., Han B.Q., Xiao X.C., Chen G., Djerdj I., Wang Y.D. (2015). Nanoparticle cluster gas sensor: Pt activated SnO_2_ nanoparticles for NH_3_ detection with ultrahigh sensitivity. Nanoscale.

[65] Zhang J., Yue D., Xia T., Cui Y., Yang Y., Qian G. (2017). A luminescent metal-organic framework film fabricated on porous Al_2_O_3_ substrate for sensitive detecting ammonia. Micropor. Mesopor. Mat..

[66] Shustova N.B., Cozzolino A.F., Reineke S., Baldo M., Dincǎ M. (2013). Selective turn-on ammonia sensing enabled by high-temperature fluorescence in metal-organic frameworks with open metal sites. J. Am. Chem. Soc..

[67] Hao J.N., Yan B. (2016). Simultaneous determination of indoor ammonia pollution and its biological metabolite in the human body with a recyclable nanocrystalline lanthanide-functionalized MOF. Nanoscale.

[68] Sousaraei A., Queirós C., Moscoso F.G., Lopes-Costa T., Pedrosa J.M., Silva A.M.G., Cunha-Silva L., Cabanillas-Gonzalez J. (2019). Subppm amine detection via absorption and luminescence turn-on caused by ligand exchange in metal organic frameworks. Anal. Chem..

[69] Lu M., Deng Y., Luo Y., Lv J., Li T., Xu J., Chen S.-W., Wang J. (2019). Graphene aerogel-metal-organic framework-based electrochemical method for simultaneous detection of multiple heavy-metal ions. Anal. Chem..

[70] Ji G.F., Liu J.J., Gao X.C., Sun W., Wang J.Z., Zhao S.L., Liu Z.L. (2017). A luminescent lanthanide MOF for selectively and ultra-high sensitively detecting Pb^2+^ ions in aqueous solution. J. Mater. Chem. A.

[71] Wang Z.J., Han L.J., Gao X.J., Zheng H.G. (2018). Three Cd (II) MOFs with different functional groups: Selective CO_2_ capture and metal ions detection. Inorg. Chem..

[72] Wang F.Q., Zhang F.X., Zhao Z.R., Sun Z.Y., Pu Y.Y., Wang Y.J., Wang X.Q. (2021). Multifunctional MOF-based probes for efficient detection and discrimination of Pb^2+^, Fe^3+^ and Cr_2_O_7_^2−^/CrO_4_^2−^. Dalton Trans..

[73] Wang Q.Y., Ke W.Q., Lou H.Y., Han Y.H., Wan J.M. (2021). A novel fluorescent metal-organic framework based on porphyrin and AIE for ultra-high sensitivity and selectivity detection of Pb^2+^ ions in aqueous solution. Dyes Pigments.

[74] Nazari M., Amini A., Eden N.T., Duke M.C., Cheng C., Hill M.R. (2021). Highly-efficient sulfonated UiO-66 (Zr) optical fiber for rapid detection of trace levels of Pb^2+^. Int. J. Mol. Sci..

[75] Shao Z., Huang C., Dang J., Wu Q., Liu Y., Ding J., Hou H. (2018). Modulation of magnetic behavior and Hg^2+^ removal by solvent-assisted linker exchange based on a water-stable 3D MOF. Chem. Mater..

[76] Wang X., Jiang Z.W., Yang C.P., Zhen S.J., Huang C.Z., Li Y.F. (2022). Facile synthesis of binary two-dimensional lanthanide metal-organic framework nanosheets for ratiometric fluorescence detection of mercury ions. J. Hazard. Mater..

[77] Moradi E., Rahimi R., Safarifard V. (2020). Porphyrinic zirconium-based MOF with exposed pyrrole Lewis base site as an efficient fluorescence sensing for Hg^2+^ ions, DMF small molecule, and adsorption of Hg^2+^ ions from water solution. J. Solid State Chem..

[78] Chen H.L., Li R.T., Wu K.Y., Hu P.P., Zhang Z., Huang N.H., Zhang W.H., Chen J.X. (2020). Experimental and theoretical validations of a one-pot sequential sensing of Hg^2+^ and biothiols by a 3D Cu-based zwitterionic metal-organic framework. Talanta.

[79] Samanta P., Desai A.V., Sharma S., Chandra P., Ghosh S.K. (2018). Selective recognition of Hg^2+^ ion in water by a functionalized metal–organic framework (MOF) based chemodosimeter. Inorg. Chem..

[80] Zhang L., Wang J., Du T., Zhang W.T., Zhu W.X., Yang C.Y., Yue T.L., Sun J., Li T., Wang J.L. (2019). NH_2_-MIL-53 (Al) Metal-organic framework as the smart platform for simultaneous high-performance detection and removal of Hg^2+^. Inorg. Chem..

[81] Khatun A., Panda D.K., Sayresmith N., Walter M.G., Saha S. (2019). Thiazolothiazole-based luminescent metal–organic frameworks with ligand-to-ligand energy transfer and Hg^2+^-sensing capabilities. Inorg. Chem..

[82] Wang H., Wang X.L., Liang M.S., Chen G., Kong R.M., Xia L., Qu F.L. (2020). A boric acid-functionalized lanthanide metal–organic framework as a fluorescence “turn-on” probe for selective monitoring of Hg^2+^ and CH_3_Hg^+^. Anal. Chem..

[83] Helal A., Naeem M., Fettouhi M., Zahir M.H. (2021). Fluorescein hydrazide-appended metal–organic framework as a chromogenic and fluorogenic chemosensor for mercury Ions. Molecules.

[84] Zhang D., Xu Y., Liu Q., Xia Z. (2018). Encapsulation of CH_3_NH_3_PbBr_3_ perovskite quantum dots in MOF-5 microcrystals as a stable platform for temperature and aqueous heavy metal ion detection. Inorg. Chem..

[85] Moradi E., Rahimi R., Farahani Y.D., Safarifard V. (2020). Porphyrinic zirconium-based MOF with exposed pyrrole Lewis base site as a luminescent sensor for highly selective sensing of Cd^2+^ and Br^−^ ions and THF small molecule. J. Solid State Chem..

[86] Mandal A., Adhikary A., Sarkar A., Das D. (2020). Naked eye Cd^2+^ ion detection and reversible iodine uptake by a three-dimensional pillared-layered Zn-MOF. Inorg. Chem..

[87] Lim K.S., Jeong S.Y., Kang D.W., Song J.H., Jo H., Lee W.R., Phang W.J., Moon D., Hong C.S. (2017). Back Cover: Luminescent metal–organic framework sensor: Exceptional Cd^2+^ turn-on detection and first in situ visualization of Cd^2+^ ion diffusion into a crystal. Chem. Eur. J..

[88] Olorunyomi J.F., Geh S.T., Caruso R.A., Doherty C.M. (2021). Metal–organic frameworks for chemical sensing devices. Mater. Horiz..

[89] Liu W., Dai X., Bai Z., Wang Y., Yang Z., Zhang L., Xu L., Chen L., Li Y., Gui D. (2017). Highly sensitive and selective uranium detection in natural water systems using a luminescent mesoporous metal–organic framework equipped with abundant lewis basic sites: A combined batch, X-ray absorption spectroscopy, and first principles simulation investigation. Environ. Sci. Technol..

[90] Liu W., Wang Y.L., Song L.P., Silver M.A., Xie J., Zhang L.M., Chen L.H., Diwu J., Chai Z.F., Wang S. (2019). Efficient and selective sensing of Cu^2+^ and UO_2_^2+^ by a europium metal-organic framework. Talanta.

[91] Wang M., Zeng G., Zhang X., Bai F.Y., Xing Y.H., Shi Z. (2021). A new family of Ln-BTC-AC-FM framework intelligent materials: Precise synthesis, structure and characterization for fluorescence detecting of UO_2_^2+^ and adsorbing dyes. J. Mol. Struct..

[92] Hou J.X., Gao J.P., Liu J., Jing X., Li L.J., Du J.L. (2019). Highly selective and sensitive detection of Pb^2+^ and UO_2_^2+^ ions based on a carboxyl-functionalized Zn (II)-MOF platform. Dyes Pigments.

[93] Chen N.N., Wang J. (2020). A serial of 2D Co-Zn isomorphous metal–organic frameworks for photodegradation and luminescent detection properties. Appl. Organometa. Chem..

[94] Guo M.Y., Li G., Yang S.L., Bu R., Piao X.Q., Guo E.Q. (2021). Metal-organic frameworks with novel catenane-like interlocking: Metal-determined photoresponse and uranyl sensing. Chem. Eur. J..

[95] Song L.P., Liu W., Wang Y.L., Chen L.H., Wang X.F., Wang S.A. (2019). A hydrolytically stable europium–organic framework for the selective detection of radioactive Th^4+^ in aqueous solution. CrystEngComm.

[96] Li J.X., Guan Q.L., You Z.X., Wang Y., Shi Z., Xing Y.H., Bai F.Y., Sun L.X. (2021). Achieving multifunctional detection of Th^4+^ and UO_2_^2+^ in the post-synthetically modified metal–organic framework and application of functional MOF membrane. Adv. Mater. Technol..

[97] Amendola V., Bergamaschi G., Boiocchi M., Alberto R., Braband H. (2014). Fluorescent sensing of ^99^Tc pertechnetate in water. Chem. Sci..

[98] Desai A.V., Sharma S., Roy A., Ghosh S.K. (2019). Probing the role of anions in influencing the structure, stability, and properties in neutral N-Donor linker based metal–organic frameworks. Cryst. Growth Des..

[99] Rapti S., Diamantis S.A., Dafnomili A., Pournara A., Skliri E., Armatas G.S., Tsipis A.C., Spanopoulos L., Malliakas C.D., Kanatzidis M.G. (2018). Exceptional TcO^4−^ sorption capacity and highly efficient ReO^4−^ luminescence sensing by Zr^4+^ MOFs. J. Mater. Chem. A.

[100] Li C.P., Zhou H., Chen J., Wang J.J., Du M., Zhou W. (2020). A highly efficient coordination polymer for selective trapping and sensing of perrhenate/pertechnetate. ACS Appl. Mater. Interfaces.

[101] Wang B., Lv X.L., Feng D.W., Xie L.H., Zhang J., Li M., Xie Y.B., Li J.R., Zhou H.C. (2016). Highly stable Zr (IV)-based metal–organic frameworks for the detection and removal of antibiotics and organic explosives in water. J. Am. Chem. Soc..

[102] Zhao D., Liu X.-H., Zhao Y., Wang P., Liu Y., Azam M., Al-Resayes S.I., Lu Y., Sun W.Y. (2017). Luminescent Cd (ii)–organic frameworks with chelating NH_2_ sites for selective detection of Fe (iii) and antibiotics. J. Mater. Chem. A.

[103] Li P., Guo M.Y., Gao L.L., Yin X.M., Yang S.L., Bu R., Gao E.Q. (2020). Photoresponsivity and antibiotic sensing properties of an entangled tris(pyridinium)-based metal–organic framework. Dalton Trans..

[104] Bai Y., Zhang M.L., Wang B.T., Ren Y.X., Zhao Y.C., Yang H., Yang X.G. (2022). Four MOFs with isomeric ligands as fluorescent probes for highly selective, sensitive and stable detection of antibiotics in water. CrystEngComm.

[105] Zhou S.H., Lu L., Liu D., Wang J., Sakiyama H., Muddassir M., Nezamzadeh-Ejhieh A., Liu J.Q. (2021). Series of highly stable Cd (ii)-based MOFs as sensitive and selective sensors for detection of nitrofuran antibiotic. CrystEngComm.

[106] Asad M., Wang S., Wang Q.Y., Li L.K., Anwar M.I., Younasc A., Zang S.Q. (2021). Aqueous media ultra-sensitive detection of antibiotics via highly stable luminescent 3D Cadmium-based MOF. New J. Chem..

[107] Gai S., Zhang J., Fan R., Xing K., Chen W., Zhu K., Zheng X.B., Wang P., Fang X.K., Yang Y.L. (2020). Highly stable zinc-based metal–organic frameworks and corresponding flexible composites for removal and detection of antibiotics in water. ACS Appl. Mater. Interfaces.

[108] Xing K., Fan R.Q., Du X., Zheng X.B., Zhou X.S., Gai S., Wang P., Yang Y.L. (2019). Dye-insertion dynamic breathing MOF as dual-emission platform for antibiotics detection and logic molecular operation. Sens. Actuators B.

[109] Xie R., Yang P., Liu J., Zou X., Tan Y., Wang X., Tao J., Zhao P. (2021). Lanthanide-functionalized metal-organic frameworks based ratiometric fluorescent sensor array for identification and determination of antibiotics. Talanta.

[110] Liu Q., Ning D., Li W.J., Du X.M., Wang Q., Li Y., Ruan W.J. (2019). Metal–organic framework-based fluorescent sensing of tetracycline-type antibiotics applicable to environmental and food analysis. Analyst.

[111] Zhou Y., Yang Q., Zhang D., Gan N., Li Q., Cuan J. (2018). Detection and removal of antibiotic tetracycline in water with a highly stable luminescent MOF. Sens. Actuators B.

[112] Li C., Yang W., Zhang X., Han Y., Tang W., Yue T., Li Z. (2020). A 3D hierarchical dual-metal–organic framework heterostructure up-regulating the pre-concentration effect for ultrasensitive fluorescence detection of tetracycline antibiotics. J. Mater. Chem. C.

[113] Zhu X.D., Zhang K., Wang Y., Long W.W., Sa R.J., Liu T.F., Lu J. (2018). Fluorescent metal–organic framework (MOF) as a highly sensitive and quickly responsive chemical sensor for the detection of antibiotics in simulated wastewater. Inorg. Chem..

[114] Zhong W.B., Li R.X., Lv J., He T., Xu M.M., Wang B., Xie L.H., Li J.R. (2020). Two isomeric In(iii)-MOFs: Unexpected stability difference and selective fluorescence detection of fluoroquinolone antibiotics in water. Inorg. Chem. Front..

[115] Vikrant K., Tsang D.C.W., Raza N., Giri B.S., Kukkar D., Kim K.H. (2018). Potential utility of metal–organic framework-based platform for sensing pesticides. ACS Appl. Mater. Interfaces.

[116] Zheng X., Zhou L., Huang Y., Wang C., Duan J., Wen L., Tian Z., Li D. (2014). A series of metal–organic frameworks based on 5-(4-pyridyl)-isophthalic acid: Selective sorption and fluorescence sensing. J. Mater. Chem. A.

[117] Kumar P., Paul A.K., Deep A. (2014). Sensitive chemosensing of nitro group containing organophosphate pesticides with MOF-5. Micropor. Mesopor. Mater..

[118] Wang H.B., Lian X., Yan B. (2020). Recyclable Eu^3+^ functionalized Hf-MOF fluorescent probe for urinary metabolites of some organophosphorus pesticides. Talanta.

[119] Ma L., He Y., Wang Y., Li R., Huang Z., Jiang Y., Gao J. (2019). Nanocomposites of Pt nanoparticles anchored on UiO66-NH_2_ as carriers to construct acetylcholinesterase biosensors for organophosphorus pesticide detection. Electrochim. Acta..

[120] Cai Y., Zhu H., Zhou W., Qiu Z., Chen C., Qileng A., Li K., Liu Y. (2021). Capsulation of AuNCs with AIE effect into metal–organic framework for the marriage of a fluorescence and colorimetric biosensor to detect organophosphorus pesticides. Anal. Chem..

[121] Luo L., Ou Y., Yang Y., Liu G., Liang Q., Ai X., Yang S., Nian Y., Su L., Wang J. (2022). Rational construction of a robust metal-organic framework nanozyme with dual-metal active sites for colorimetric detection of organophosphorus pesticides. J. Hazard. Mater..

[122] Wiwassuku T., Boonmak J., Burakham R., Hadsadee S., Jungsuttiwong S., Bureekaew S., Promarak V., Youngme S. (2021). Turn-on fluorescent probe towards glyphosate and Cr^3+^ based on Cd(ii)-metal organic framework with Lewis basic sites. Inorg. Chem. Front..

[123] Li Y., Wu S., Zhang Y., Ma Z., Zhu M., Gao E. (2021). A lanthanide metal–organic framework as ratio fluorescence probe to detect pesticides in water. Inorg. Chim. Acta.

[124] Chen H., Fan P., Tu X., Min H., Yu X., Li X., Zeng J.L., Zhang S., Cheng P. (2019). A bifunctional luminescent metal–organic framework for the sensing of paraquat and Fe^3+^ ions in water. Chem. Asian J..

[125] Parmar B., Bisht K.K., Rachuri Y., Suresh E. (2020). Zn (II)/Cd (II) based mixed ligand coordination polymers as fluorosensors for aqueous phase detection of hazardous pollutants. Inorg. Chem. Front..

[126] Zhao B., Yang Q., Wang J.S., Xie F.Y., Yu H.Y., Li Y., Ma Y.X., Ruan W.J. (2021). An anionic-ligand installed pyrene-based MOF for the fluorescence detection of paraquat. New J. Chem..

[127] Wei Z., Chen D., Guo Z., Jia P., Xing H. (2020). Eosin Y-embedded zirconium-based metal–organic framework as a dual-emitting built-in self-calibrating platform for pesticide detection. Inorg. Chem..

[128] Sun B., Tao T., Liu L., Ding R., Mao Y. (2021). Electron transfer facilitated by π–π stacking during the nitrobenzene recognition process of an MOF Sensor. J. Phys. Chem. C.

[129] Qin J.H., Huang Y.D., Shi M.Y., Wang H.R., Han M.L., Yang X.G., Li F.F., Ma L.F. (2020). Aqueous-phase detection of antibiotics and nitroaromatic explosives by an alkali-resistant Zn-MOF directed by an ionic liquid. RSC Adv..

[130] Wang X.S., Lia L., Yuan D.Q., Huang Y.B., Cao R. (2018). Fast, highly selective and sensitive anionic metal-organic framework with nitrogen-rich sites fluorescent chemosensor for nitro explosives detection. J. Hazard. Mater..

[131] Lan A., Li K., Wu H., Olson D.H., Emge T.J., Ki W., Hong M., Li J. (2009). A luminescent microporous metal–organic framework for the fast and reversible detection of high explosives. Angew. Chem. Int. Ed..

[132] Pramanik S., Zheng C., Zhang X., Emge T.J., Li J. (2011). New microporous metal−organic framework demonstrating unique selectivity for detection of high explosives and aromatic compounds. J. Am. Chem. Soc..

[133] Nagarkar S.S., Joarder B., Chaudhari A.K., Mukherjee S., Ghosh S.K. (2013). Highly selective detection of nitro explosives by a luminescent metal–organic framework. Angew. Chem. Int. Ed..

[134] Li J., Wang J., Li H., Song N., Wang D., Tang B.Z. (2020). Supramolecular materials based on AIE luminogens (AIEgens): Construction and applications. Chem. Soc. Rev..

[135] Guo Y., Feng X., Han T., Wang S., Lin Z., Dong Y., Wang B. (2014). Tuning the luminescence of metal–organic frameworks for detection of energetic heterocyclic compounds. J. Am. Chem. Soc..

[136] Wang Y.N., Wang S.D., Chang X.P., Li H.F., Zhang J.M., Xu L., Wang S.Y. (2021). A new fluorescence MOF for highly sensitive detection of acetylacetone. ChemistrySelect.

[137] Su Y.Q., Qu Y.H., Fu L., Gui G.H. (2020). An unprecedented binodal (4,6)-connected Co (II) MOF as dual-responsive luminescent sensor for detection of acetylacetone and Hg^2+^ ions. Inorg. Chem. Commun..

[138] Lu Z.Z., Zhang R., Li Y.Z., Guo Z.J., Zheng H.G. (2011). Solvatochromic behavior of a nanotubular metal−organic framework for sensing small molecules. J. Am. Chem. Soc..

[139] Li B., Chen X., Hu P., Kirchon A., Zhao Y.M., Pang J., Zhang T., Zhou H.C. (2019). Facile fabrication of a multifunctional metal–organic framework-based sensor exhibiting exclusive solvochromic behaviors toward ketone molecules. ACS Appl. Mater. Interfaces.

[140] Li Y., An J.D., Wang T.T., Shi Y.F., Huo J.Z., Wu X.X., Liu Y.Y., Ding B. (2021). An ultra-stable Cadmium (II) coordination framework constructed from the new bi-functional ligand and application as fluorescent probe for acetylacetone and antibiotics. Dyes Pigments.

[141] Ghosh S., Das A., Biswas S. (2021). A functionalized UiO-66 MOF acting as a luminescent chemosensor for selective and sensitive turn-on detection of superoxide and acetylacetone. Micropor. Mesopor. Mater..

[142] Kang X.M., Fan X.Y., Hao P.Y., Wang W.M., Zhao B. (2019). A stable zinc–organic framework with luminescence detection of acetylacetone in aqueous solution. Inorg. Chem. Front..

[143] Li H.Y., Zhao S.N., Zang S.Q., Li J. (2020). Functional metal–organic frameworks as effective sensors of gases and volatile compounds. Chem. Soc. Rev..

[144] Kumar P., Kim K.H., Rarotra S., Ge L., Lisak G. (2020). The advanced sensing systems for NO_x_ based on metal-organic frameworks: Applications and future opportunities. Trends Anal. Chem..

[145] Mallick A., Garai B., Addicoat M.A., Petkov P.S., Heine T., Banerjee R. (2015). Solid state organic amine detection in a photochromic porous metal organic framework. Chem. Sci..

[146] Mallick A., El-Zohry A.M., Shekhah O., Yin J., Jia J., Aggarwal H., Emwas A.H., Mohammed O.F., Eddaoudi M. (2019). Unprecedented ultralow detection limit of amines using a thiadiazole-functionalized Zr (IV)-based metal–organic framework. J. Am. Chem. Soc..

